# Recent Advances in BTK Inhibitors for the Treatment of Inflammatory and Autoimmune Diseases

**DOI:** 10.3390/molecules26164907

**Published:** 2021-08-13

**Authors:** Datong Zhang, He Gong, Fancui Meng

**Affiliations:** 1 School of Chemistry and Chemical Engineering, Qilu University of Technology (Shandong Academy of Sciences), 3501 Daxue Road, Jinan 250353, China; gonghedeyouxiang@163.com; 2 Tianjin Key Laboratory of Molecular Design and Drug Discovery, Tianjin Institute of Pharmaceutical Research, 306 Huiren Road, Tianjin 300301, China; mengfc@tjipr.com

**Keywords:** BTK inhibitors, autoimmune, inflammation, treatment, structural analysis

## Abstract

Bruton’s tyrosine kinase (BTK) plays a crucial role in B-cell receptor and Fc receptor signaling pathways. BTK is also involved in the regulation of Toll-like receptors and chemokine receptors. Given the central role of BTK in immunity, BTK inhibition represents a promising therapeutic approach for the treatment of inflammatory and autoimmune diseases. Great efforts have been made in developing BTK inhibitors for potential clinical applications in inflammatory and autoimmune diseases. This review covers the recent development of BTK inhibitors at preclinical and clinical stages in treating these diseases. Individual examples of three types of inhibitors, namely covalent irreversible inhibitors, covalent reversible inhibitors, and non-covalent reversible inhibitors, are discussed with a focus on their structure, bioactivity and selectivity. Contrary to expectations, reversible BTK inhibitors have not yielded a significant breakthrough so far. The development of covalent, irreversible BTK inhibitors has progressed more rapidly. Many candidates entered different stages of clinical trials; tolebrutinib and evobrutinib are undergoing phase 3 clinical evaluation. Rilzabrutinib, a covalent reversible BTK inhibitor, is now in phase 3 clinical trials and also offers a promising future. An analysis of the protein–inhibitor interactions based on published co-crystal structures provides useful clues for the rational design of safe and effective small-molecule BTK inhibitors.

## 1. Introduction

Bruton’s tyrosine kinase (BTK) is a nonreceptor cytoplasmic tyrosine kinase in the Tec family of protein tyrosine kinases. BTK was first identified in the primary immunodeficiency disease X-linked agammaglobulinemia in humans, which is caused by a BTK gene mutation [1]. BTK is expressed in all hematopoietic cells except T cells, natural killer cells and plasma cells. In B lymphocytes, BTK activity is essential in B-cell receptor (BCR)-mediated activation, leading to cell development, antibody and cytokine production, and costimulatory molecule expression [2,3,4]. Aberrant activation of B cells is demonstrated to play a central role in the pathogenesis of B-cell malignancies, autoimmune diseases and inflammation [5,6]. Great success has been achieved in the development of covalent irreversible BTK inhibitors for the treatment of hematological malignancies, as exemplified by ibrutinib, the first effective and selective BTK inhibitor approved by the Food and Drug Administration (FDA) in 2013 to control a variety of B-cell tumors [5,7,8,9,10]. BTK is also involved in the activation of innate immune cells, including macrophages, neutrophils, mast cells and basophils. BTK positively regulates Fcγ receptor (FcγR) signaling in macrophages and Fcε receptor (FcεR) signaling in mast cells and basophils. When BTK is activated through the Fcγ and Fcε receptors, downstream signaling induces the expression of pro-inflammatory cytokines, chemokines, and cell adhesion molecules [11,12]. Toll-like receptors (TLRs) are a primary surveillance system that can recognize pathogen- and damage-associated molecular patterns (PAMP and DAMP), which are important to the activation of host defense [13]. BTK is required for the production of TLRs-induced pro-inflammatory cytokines; however, the relationships between BTK and different TLRs are not fully understood [14]. Chemokines are associated with a number of diseases, including chronic inflammation [15], autoimmune disease [16], cancer [17], and so on. BTK also participates in chemokine receptor signal transduction. BTK inhibition by ibrutinib suppressed CXC-chemokine ligand 12 (CXCL12), CXCL13, and CC-chemokine ligand 19 (CCL19)-induced signaling, adhesion, and the migration of primary chronic lymphocytic leukemia (CLL) cells [17], and also led to reduced surface membrane levels of CXC-chemokine receptor 4 (CXCR4) and impaired CXCR4 function [18]. It was demonstrated recently that inhibition of CXCL12 provided a significant anti–inflammatory effect in a murine allergic model [19]. Given the central role of BTK in BCR and FcR signaling pathways as well as its important function in the regulation of TLRs and chemokine receptors, inhibition of BTK represents an attractive potential therapeutic approach for the treatment of autoimmune and inflammatory disorders in which B lymphocytes and myeloid cells induce or sustain an excessive autoimmune response. 

For over a decade, there have been great efforts devoted to developing BTK inhibitors for potential clinical application in chronic inflammatory diseases and autoimmune diseases, in addition to hematologic malignancies [8,9,10]. Studies demonstrated that B cells are crucial players in inflammatory and autoimmune diseases including rheumatoid arthritis (RA), systemic lupus erythematosus (SLE), Sjogren’s syndrome (SS), and multiple sclerosis (MS) [20,21,22,23]. These diseases are marked by altered B cell selection resulting in the production of autoreactive antibodies and pro-inflammatory cytokines. BTK-deficient mice were found to be protected from SLE and autoimmune arthritis [24]. BTK inhibition in experimental rodent models led to amelioration of the SLE associated manifestations, including nephritis, cutaneous and neuropsychiatric diseases [25,26]. RA is a multifactorial autoinflammatory disease that is characterized by synovial membrane hyperplasia with infiltration of inflammatory cells, including B cells, T cells and macrophages. Increased BTK protein and phosphorylation were observed in the peripheral blood B cells from patients with RA compared with healthy controls [27]. The importance of FcγRs in RA pathogenesis was also demonstrated [28]. As a potent BTK inhibitor, ibrutinib exhibited therapeutic effects in both the collagen-induced arthritis (CIA) and collagen antibody-induced arthritis (CAIA) rodent models of RA in a dose-dependent manner [29,30,31]. It also showed an inhibitory effect on FcγR- and FcεR-mediated cytokine release by monocytes, macrophages and mast cells [31]. BTK inhibition also led to positive outcomes in some other autoimmune diseases, including urticaria (NCT03137069), immune thrombocytopenia (ITP) (NCT04562766), pemphigus (NCT03762265), and IgG4-related disease (RD) (NCT04520451). Recent clinical results demonstrated the effectiveness of BTK inhibitors in treating excessive inflammation and protecting against severe lung injury in the context of severe Coronavirus Disease 19 (COVID-19) [32]. A number of BTK inhibitors have shown efficacy in several preclinical models of inflammatory or autoimmune diseases that are driven by pathogenic B cells or myeloid cells and several molecules have progressed into clinical trials, but thus far there are no approved BTK inhibitors for inflammatory or autoimmune disorders. 

Small-molecule BTK inhibitors have great potential as therapeutics to treat inflammatory or autoimmune diseases and as tools to probe the biology of BTK. There have been several excellent reviews in this field so far. Most of them mainly described the development of BTK inhibitors in treating RA [33,34,35] or discussed the validity of BTK inhibition in autoimmune diseases [6,36]. This review will cover recent advances in the field of medicinal chemistry towards small-molecule BTK inhibitors under clinical trials for the treatment of inflammatory and autoimmune diseases, including RA, SS, MS, SLE, urticaria, pemphigus, ITP, and RD. The diverse BTK inhibitors in preclinical development were also included. This review provides a summary of the inhibitor-protein interactions based on the available structural data, therefore supplying useful clues for the rational design and optimization of potent and specific small-molecule BTK inhibitors. Additionally, a comparison of the three design strategies demonstrates the good prospect of irreversible covalent and reversible covalent BTK inhibitors.

## 2. BTK as a Biological Target for Drug Development

### 2.1. BTK Structure and Biological Functions

**Figure 1:**
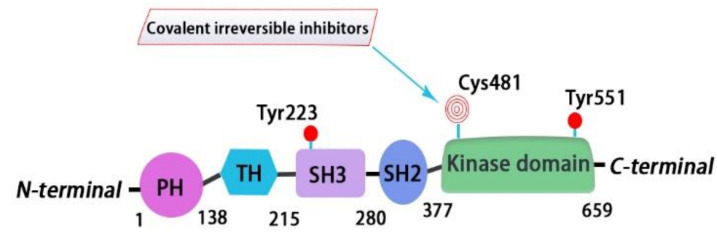
Bruton’s tyrosine kinase (BTK) structure diagram. BTK is composed of 659 amino acids and contains five domains. From N-terminal to C-terminal, domains are listed as the pleckstrin homology (PH) domain, proline-rich TEC homology (TH) domain, SRC homology domains SH3 and SH2, and the catalytic kinase domain. Currently, six approved BTK inhibitors target the kinase domain of BTK, forming a covalent bond with Cys481.

**Figure 2:**
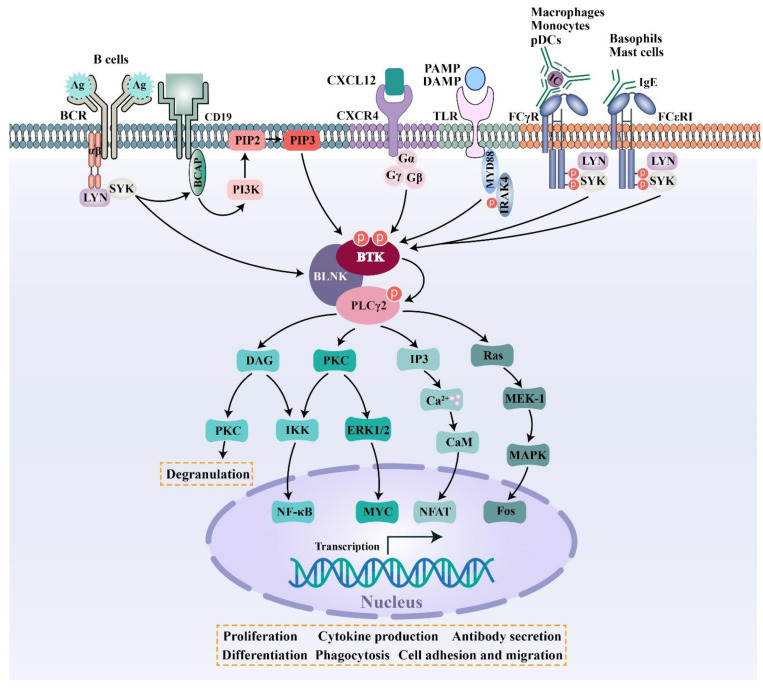
BTK regulates multiple receptor signaling pathways including those of the B-cell receptor (BCR), Fc receptor (FcR), Toll- like receptor (TLR) and chemokine receptor.

BTK is a 659-amino-acid protein and mainly contains five domains, including a N-terminal pleckstrin homology (PH) domain, a proline-rich TEC homology (TH) domain, two SRC homology domains (SH2/SH3), and a C-terminal tyrosine kinase domain (TK) (also known as SRC homology 1 or SH1) [37]. The PH domain has been shown to bind to phosphatidylinositol 3,4,5-triphosphate (PIP3) and promote the localization of BTK to the cell membrane. The TH domain contains a highly conserved zinc-finger motif and a proline-rich region. The first transphosphorylation occurs at Tyr551 in the catalytic kinase domain by LYN or spleen tyrosine kinase (SYK). Then, autophosphorylation at Tyr223 within the SH3 domain stabilizes the active conformation and fully activates BTK activity. The activated BTK will then interact with the adapter protein BLNK in the SH2 domain and activate a series of downstream cascade reactions [4] (Figure 1 and Figure 2). 

BTK is a key kinase that interconnects BCR signaling, FcR signaling, TLR signaling, and chemokine receptor signaling. BCR is an antigen-specific receptor expressed on the B cell surface. BCR is always coupled with the Igα/Igβ heterodimer, which is incorporated into the BCR complex. When specific antigens bind to the BCR complex, the SRC family kinase LYN phosphorylates the immunoreceptor tyrosine-based activation motif (ITAMs) residues within the cytoplasmic tails of Igα/Igβ, followed by the activation of SYK. Once activated, SYK phosphorylates a B-cell linker protein termed BLNK, which then acts as a bridge interfacing BTK and phospholipase C-γ2 (PLCγ2). In the macro-molecular complex, BTK is activated by successive phosphorylation on two key tyrosine residues (Tyr551 and Tyr223). Subsequently activated BTK and SYK phosphorylate PLCγ2. Further downstream signaling results in the activation of the NFAT, NF-κB, and MAPK pathways, etc, leading to cell proliferation and differentiation, antibody and cytokine production, and costimulatory molecule expression [5,38] (Figure 2). Two of the major integrins that are expressed on the surface of B cells—that is, lymphocyte function-associated antigen-1 (LFA-1) and very late antigen 4 (VLA-4)—are both important in mediating the adhesion and migration of B cells. LTA-1 and VLA-4 can be activated via inside-out signaling by BCR triggering in B cells. In the process of inside-out activation, both LTA-1 and VLA-4 require the activation of SRC-family kinases and phosphoinositide 3-kinase (PI3K) [39,40]. VLA-4 integrin plays a pivotal role in mediating both cell–cell and cell–matrix interactions in CLL-involved tissues by interacting with vascular cell adhesion molecule 1 (VCAM-1) and fibronectin, respectively. It was demonstrated that the VLA-4, expressed by CD49d-positive CLL, can still be inside-out activated upon BCR stimulation, leading to the increased adhesive capacities of CLL cells. Although the constitutive VLA-4 activation and cell adhesion were reduced, BCR-mediated VLA-4 activation is retained in ibrutinib-treated CLL cells, demonstrating that the inhibition of BTK activity is not sufficient to block BCR-dependent VLA-4 activation. Noteworthy, a concomitant inhibition of both BTK and PI3K was able to completely abrogate the integrin response to BCR stimulation [40].

Different isotypes of immunoglobulins act as effectors, partly via binding to the respective FcRs. IgG antibodies bind to FcγR on macrophages, monocytes, and plasmacytoid dendritic cells (pDCs) to initiate cellular signaling pathways, which ultimately trigger cellular activation, inflammatory cytokine production or phagocytosis [41]. Most FcγRs can only interact with antibodies in the form of immune complexes (IC), leading to high-avidity binding [41]. IgE antibodies bind to FcεR on mast cells and basophils to induce degranulation and cytokine production [12,42]. BTK is activated after receptor activation, and then triggers downstream signaling events. 

In addition to B and T lymphocyte cells, 10 TLRs identified in humans have been found to be variably expressed on many different cell types, including macrophages, monocytes, neutrophils, dendritic cells, and endothelial cells [13]. Most TLRs are expressed on the cell surface and recognize invading pathogens outside of the cell. A set of TLRs, consisting of TLR3, TLR7, TLR8 and TLR9, are expressed intracellularly in endosomes and recognize nucleic acid derived from various sources, including viruses, bacteria or DAMPs [13]. In antigen-independent TLR signaling pathways, most TLRs require MYD88 to mediate activation signals. BTK is recruited to a complex and further downstream signaling promotes cell proliferation, antibody secretion, and the production of pro-inflammatory cytokines [14]. However, the function of BTK in different TLRs still needs to be further explored [14,43] (Figure 2).

BTK is also involved in chemokine receptor signal transduction. CXCL12 is highly expressed in the bone marrow and germinal centers. CXCL12 can couple with CXCR4 and then activate BTK through direct interactions between BTK and the CXCR4-linked heterotrimeric G protein subunits. Next, activated BTK phosphorylates downstream PLCγ2, ERK1/2, JNK, and AKT, thus modulating the migration and adhesion of B cells (Figure 2) [38].

### 2.2. Analysis of Ligand–Protein Interactions between BTK and Representative Inhibitors

All kinases transfer phosphate groups from ATP to a substrate. The ATP binding site is situated in the catalytic kinase domain. As with other kinases, the N- and C-terminal lobes in the catalytic domain SH1 of BTK form the walls of a deep cavity, which allocates the coenzyme ATP in a pocket called ATP-binding site. Although, to date, no crystal structure of the BTK-ATP complex has been disclosed, the available data from the co-crystal structures of some inhibitors bound to BTK enzyme indicate the key interactions between ATP and the binding site [28,44]. ATP binds in the pocket with the adenine ring, making hydrogen bonds with the BTK backbones of Met477 and Glu475. These two residues are localized in the segment that connects the N- and C-lobes, generally called the hinge region. To mimic the intermolecular interactions between ATP adenine and the hinge region, all BTK inhibitors possess a heterocycle core to maintain similar interactions with hinge residues. 

**Figure 3:**
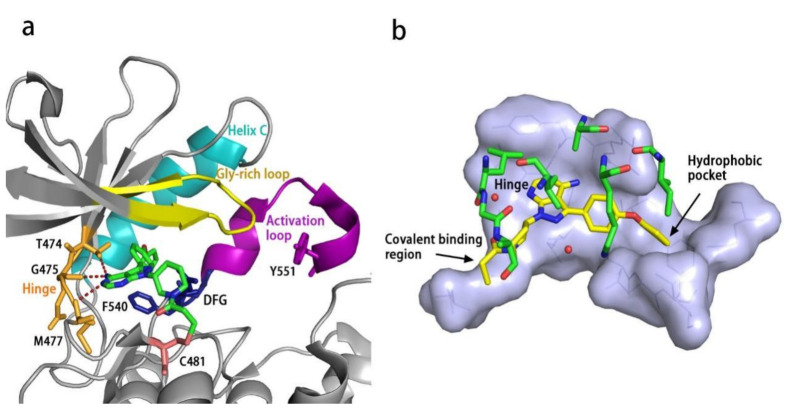
(**a**) X-ray crystal structure of ibrutinib-BTK complex in cartoon representation (PDB code: 5P9J). Ibrutinib is shown as sticks and the carbon atoms are depicted in green. (**b**) Illustration of the location of key regions of BTK when bound to inhibitor ibrutinib. Ibrutinib is shown as sticks and the carbon atoms are depicted in yellow. Surface view of the binding site is composed by the residues within 5 Å of ibrutinib and some residues in front of the ligand are shown in green sticks for clarity. All structural images were created using PyMoL [45].

**Figure 4:**
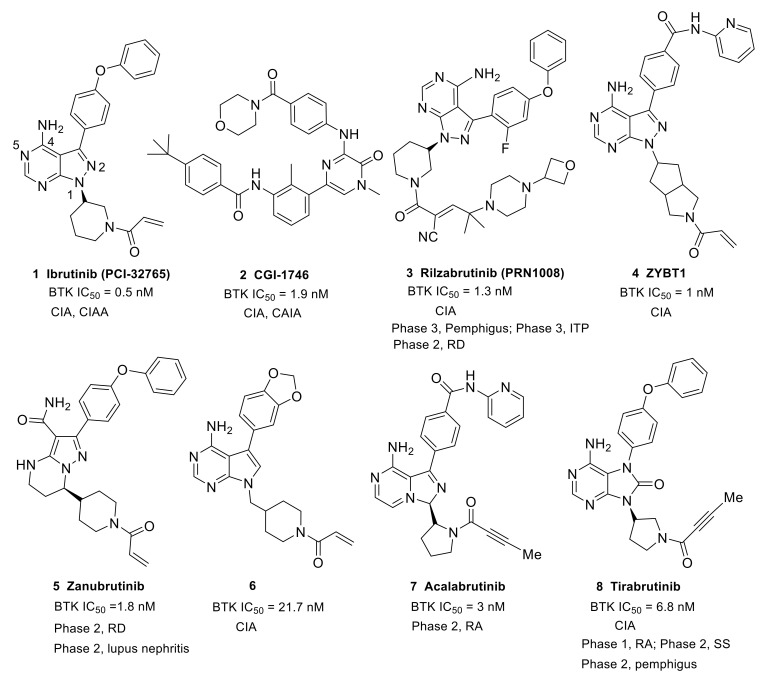
The representative structures of three types BTK inhibitors and some covalent irreversible inhibitors with fused bicyclic heterocycle scaffolds and their progress in the treatment of autoimmune and inflammatory diseases. CIA, collagen-induced arthritis; CAIA, collagen antibody-induced arthritis; ITP, immune thrombocytopenia; RD, IgG4-related disease; RA, rheumatoid arthritis; SS, Sjogren’s syndrome.

Ibrutinib (**1**) (PCI-32765) is an orally bioavailable and covalent, irreversible and ATP-competitive inhibitor (Figure 3 and Figure 4 [29]. The co-crystal structure analysis revealed that 4-amino and 5-nitrogen on the pyrazolopyrimidine ring each formed one hydrogen bond with Glu475 and Met477 in the hinge region, respectively, and the 4-amino group was also within H-bonding distance to Thr474 side-chain alcohol (gatekeeper residue in the hinge region). Side chains from Val416 in the glycine-rich P-loop, Tyr476 in the hinge, as well as residues Ala428 and Leu528, encircled the pyrazolopyrimidine moiety at a distance of interaction. A water-mediated hydrogen bond was formed between the N2 of pyrazolopyrimidine and the NH_2_ of Lys430. The terminal phenyl group inserted into a hydrophobic pocket to form π-π stacking with the DFG residue Phe540 at the bottom. The diphenyl ether moiety was also surrounded by αC helix (Met449), DFG motif (Asp539), activation loop (Leu542) and residues Lys430, Ser538, Ile472 and Thr474. The partially solvent-exposed N-acryloyl piperidine moiety was located at the opening of the active site and sandwiched between Leu408 and the Gly480-Cys481 motif. The electrophilic acrylamide covalently linked to the thiol group of Cys481 residue at the rim of the pocket. The acrylamide carbonyl oxygen formed a direct hydrogen bond with the backbone NH of Cys481 and was simultaneously involved in a water-mediated intramolecular hydrogen bond with the N7 of pyrazolopyrimidine. It was found that ibrutinib-bound BTK kinase domain adopted an inactive conformation—that is, the activation loop was collapsed into the active site and the αC-helix was out [44].

**Figure 5:**
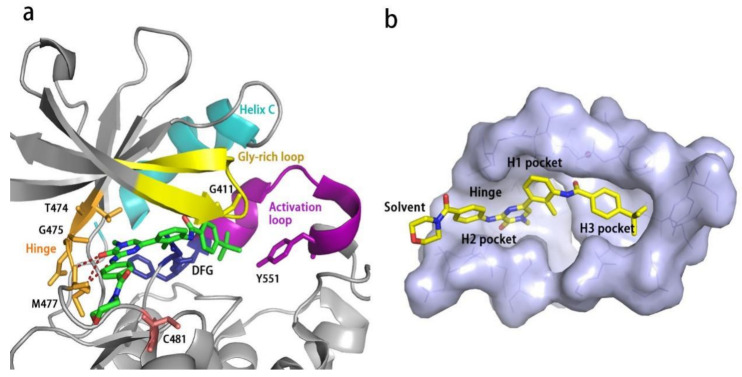
(**a**) X-ray crystal structure of CGI-1746 bound in BTK (PDB code: 3OCS) in cartoon representation. CGI-1746 is shown as sticks and the carbon atoms are depicted in green. (**b**) Illustration of the location of key regions of BTK when bound to inhibitor CGI-1746. Surface view of the binding site is composed by the residues within 5 Å of CGI-1746. All structural images were created using PyMoL [45].

Similar to other kinases, the binding pocket of BTK inhibitors has an impressive ability to accommodate a multitude of diverse small molecules. CGI-1746 (**2**) is a non-covalent, selective, reversible BTK inhibitor and exhibited different binding modes with the enzyme relative to ibrutinib (Figure 4 and Figure 5). Co-crystal structure revealed that CGI-1746 bound to BTK in an extended conformation, resulting in the formation of a large, narrow binding pocket. The central phenyl ring of CGI-1746 occupied a shallow, hydrophobic subpocket (H1 pocket) lined by Leu408, Gly409, Thr410 and Val416 of the Gly-rich P-loop. The t-butylphenyl moiety fit in the lipophilic H3 pocket that was quite deep in the active site. The tert-butyl group was oriented towards the bottom of the H3 region formed by Leu542, Ser543, Val546, Tyr551 (from A-loop) and Asp521. The t-butylphenyl group was surrounded by Gly-rich P-loop residues Gln412 and Phe413, DFG residue Asp539 and catalytic loop residue Asn526, at the periphery of the pocket. The carbonyl oxygen of t-butylphenyl amide formed a direct hydrogen bond with the NH_2_ of Lys430 as well as a water-mediated hydrogen bond with the backbone NH of Gly411 (P-loop). In the H2 pocket, the amino and carbonyl oxygen on the pyrazinone ring bound to the hinge residue Met477 to make two hydrogen bonds; meanwhile, the dipolar interactions between the N-methyl and backbone carbonyl of Glu475 (hinge residue) and the side chain hydroxyl group of gatekeeper Thr474 were observed. In addition to the three residues mentioned above, Leu408, Ala428, Val458, Tyr476, Ala478, Asn479, Gly480 and Leu528 contributed to the establishment of H2 pocket to accommodate the aminopyrazinone and, partly, the phenyl ring attached on the secondary amine. The morpholine-amide segment extended into the solvent area. Crystallographic analysis showed that CGI-1746 stabilized the inactive DFG-in conformation [28]. 

## 3. Current Development of BTK Inhibitors for the Treatment of Autoimmune and Inflammatory Diseases

**Figure 6:**
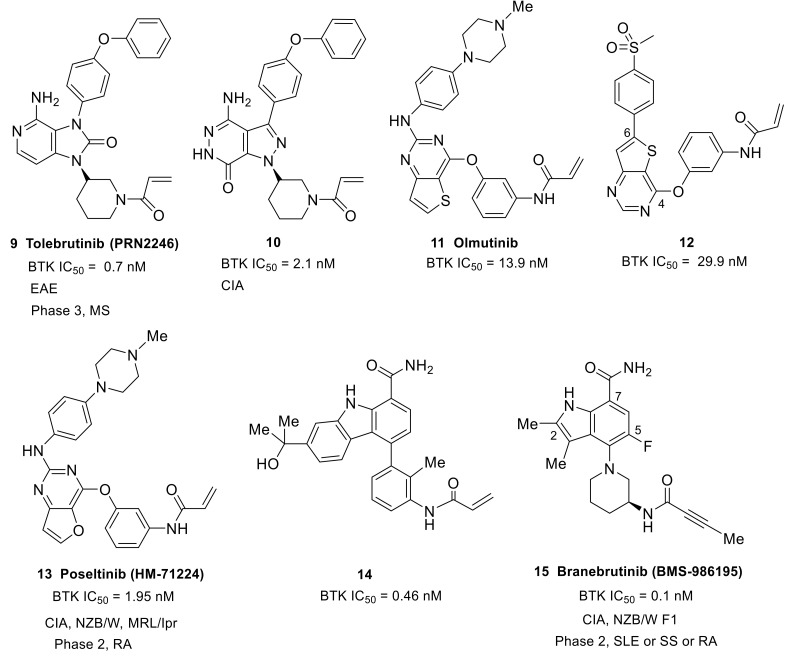
The representative structures of covalent irreversible BTK inhibitors with fused bicyclic heterocycle scaffolds and their progress in the treatment of autoimmune and inflammatory diseases. EAE, experimental autoimmune encephalomyelitis; MS, multiple sclerosis; SLE, systemic lupus erythematosus.

**Figure 7:**
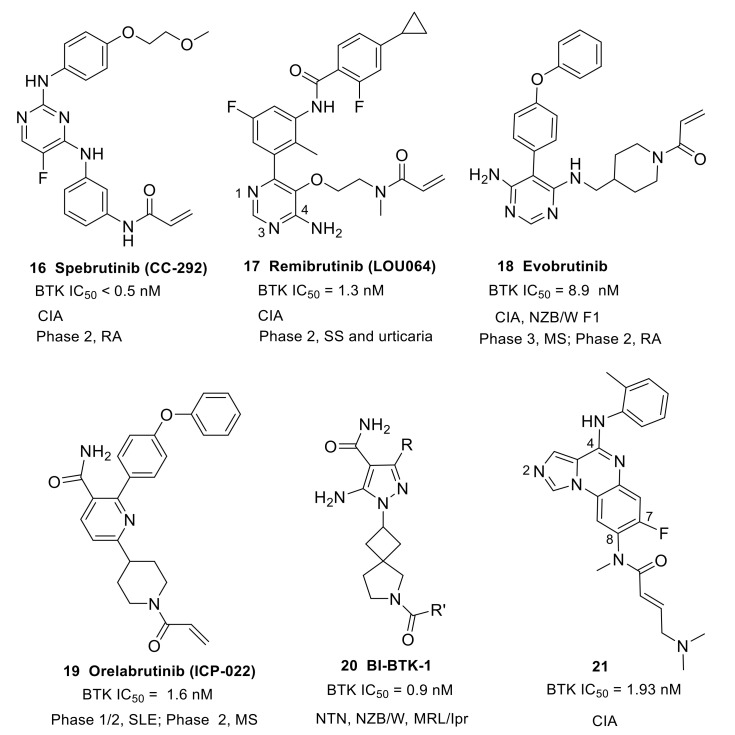
The representative structures of covalent irreversible BTK inhibitors with monoheterocycle and fused tricyclic heterocycle scaffolds and their progress in the treatment of autoimmune and inflammatory diseases. NTN, nephrotoxic nephritis.

The essential role of BTK in autoimmune and chronic inflammatory conditions prompted the development of small-molecule BTK inhibitors. To date, the most established small-molecule BTK inhibitors can be divided into two types: covalent irreversible inhibitors and the non-covalent reversible inhibitors. Covalent inhibitors feature an α,β-unsaturated amide as an electrophilic warhead which generally targets Cys481 in the ATP-binding pocket of BTK to form a covalent bond via a Michael addition reaction. Non-covalent inhibitors that do not possess a Michael addition acceptor mainly access a specific pocket (H3 pocket) under the inactive conformation of BTK. Irreversible inhibitors have the advantage of longer-lasting inhibitory activity, but their binding mode is restricted by the need to react with Cys481 residue. Comparatively speaking, non-covalent inhibitors are freer to contact different regions within the active site. Rilzabrutinib (**3**) (PRN1008), with a cyanoacrylamide electrophile, was demonstrated to be a reversible covalent BTK inhibitor that showed prolonged and tunable residence time [46] (Figure 4). This provides a third type of inhibitors. Three types of BTK inhibitors will be discussed in this review. All the inhibitors possess a core heterocycle moiety, which can form essential hydrogen bonds with the hinge region of BTK. These inhibitors are further categorized based on their core heterocycle scaffolds. All six approved BTK inhibitors are covalent, irreversible inhibitors that all possess an α,β-unsaturated amide electrophile (Figure 4, Figure 6 and Figure 7). For the approved BTK inhibitors, we will first briefly introduce their information and then focus on the recent efforts in the treatment of autoimmune and inflammation diseases. For the compounds that are under investigation at the preclinical and clinical stages, the biological activity, animal model, structure–activity relationships (SAR) and interaction mode with the binding pocket (if any) will be given primary focus. A table which summarized all BTK inhibitors with IC_50_, clinical stage, administration route, dosage form, and (proposed) dose was put in the Supporting Information (Table S1).

### 3.1. Covalent Irreversible Inhibitors

This type of compound contains a central heterocycle core, flanked by an electrophilic warhead and a hydrophobic group, which are connected to the core via lipophilic spacers, respectively. The electrophilic warhead is either an acrylamide or an alkynyl amide, which should only react and form a covalent bond with Cys481. 

#### 3.1.1. Fused Bicyclic Heterocycle (Pyrazolopyrimidine, Pyrrolopyrimidine, Imidazopyrazine, Purinone, Imidazolonepyridine, Pyrazolopyridazinone, Thienopyrimidine, Furopyrimidine, and Indole) 

Ibrutinib (**1**) (PCI-32765), originally identified by Pharmacyclics and developed jointly with Johnson, is the first-in-class covalent irreversible BTK inhibitor (Table 1, Figure 4). In ibrutinib’s structure, a diphenyl ether and an N-acryloyl piperidine moiety were joined by a pyrazolopyrimidine core. Ibrutinib was first launched in the U.S. in 2013 to treat mantle cell lymphoma (MCL) and was then approved in different countries for the treatment of CLL [47], Waldenstrom’s macroglobulinemia (WM) [48] and chronic graft-versus-host disease (cGVHD) [49]. In addition, ibrutinib showed therapeutic effects in both the CIA and CAIA animal models of RA as well as suppressive effects on the FcεR- and FcγR-mediated cytokine release by monocytes, macrophages and mast cells. Ibrutinib is highly potent against BTK, with an IC_50_ of 0.5 nM; however, 0.3- to 4-fold unsatisfactory selectivity ratios over BLK, BMX, TXK and HCK kinases were observed. Ibrutinib also demonstrated moderate to high potency towards hERG, JAK3, EGFR, ERBB4, and TEC [50,51]. Therefore, side effects such as rash, atrial fibrillation, diarrhea, and bleeding have emerged due to the off-target effects of ibrutinib [52,53]. Ibrutinib is not approved by the FDA for the treatment of RA, SLE and other autoimmune diseases owing to toxicities related to off-target effects.

Recently, Indian researchers developed ZYBT1 (**4**) as a covalent irreversible, specific BTK inhibitor that inhibited the ibrutinib-resistant C481S BTK with nanomolar potency (Figure 4). Structurally, it has a pyrazolo[3,4-d]pyrimidine core and the acryloyl group was incorporated via a hexahydrocyclopenta[c]pyrrole linker. ZYBT1 had potent inhibitory activity for wild-type as well as C481S mutants of BTK, with IC_50_ values of 1 and 14 nM, respectively. ZYBT1 blocked the phosphorylation of BTK-Y223 and PLCγ2 and inhibited the secretion of TNF-α, IL-8 and IL-6 (IC_50_: 1.0, 0.4, and 0.2 nM for TNF-α, IL-6 and IL-8, respectively). In a screening test of 13 different kinases, ZYBT1 demonstrated high selectivity for BTK and TEC, while for other tyrosine kinases, namely EGFR, JAK3, BLK, ITK and ERB, ZYBT1 was >60-fold less potent. This compound showed robust efficacy in murine CIA models and a streptococcal cell wall (SCW)-induced model and almost complete remission was demonstrated after 20 days of once daily treatment. In fact, 0.6 mg/kg ZYBT1 provided comparable efficacy as 3 mg/kg ibrutinib in CIA model. The favorable pharmacokinetic properties made ZYBT1 suitable for the use as an oral anti-arthritic and anti-cancer drug [54].

In order to improve the aqueous solubility and pharmacokinetic (PK) properties of BTK inhibitors, Chen’s group optimized the structure of ibrutinib to afford a series of compounds bearing the 7*H*-pyrrolo [2,3-d]pyrimidin-4-amine scaffold that potently inhibited BTK in vitro [50]. The SAR work revealed that replacement of the 4-phenoxyphenyl on the C3-position of pyrryl with benzo[d] [1,3]dioxol-5-yl moiety enhanced the aqueous solubility significantly (about 10-fold increase), which resulted in the discovery of the optimal compound **6** (Figure 4). Compound **6** preferentially inhibited BTK (IC_50_ = 21.7 nM) over closely related kinases with moderate selectivity and had no inhibition to EGFR, ErbB2, ErbB4, Itk or JAK3 (IC_50_ > 10 μM). Additionally, the compound showed a markedly weaker potential to block the hERG channel (IC_50_ = 11.10 μM) relative to ibrutinib (IC_50_ = 0.97 μM), which was indicative of a low risk for cardiotoxicity. The analysis of hydrophobic interactions in a docking model between BTK and compound **6** explained that the slight decrease in potency of the compound could be attributed to the fact that benzo [d] [1,3]dioxol-5-yl cannot completely fill the hydrophobic pocket, as well as its weaker hydrophobicity than phenoxyphenyl group. Cell-based tests revealed that compound **6** markedly inhibited BTK-Y223 autophosphorylation and PLCγ2-Y1217 phosphorylation. MTT revealed that compound **6** demonstrated low cytotoxicity towards LO2, HEK293 and THP-1 cell lines. Due to its favorable physicochemical properties (ClogP = 2.53, aqueous solubility ≈ 0.1 mg/mL) and PK profiles (F = 49.15%, t_1/2_ = 7.02 h), compound **6** showed a potent anti-arthritis activity and similar efficacy to ibrutinib in reducing paw thickness in a CIA mice model. Collectively, compound **6** was a potent, selective and durable inhibitor of BTK and had the potential to be a safe and efficacious treatment for arthritis [50].

Acalabrutinib (**7**) (ACP-196) is a second-generation irreversible BTK inhibitor developed by Acerta Pharma. It has an imidazopyrazinamine core (Figure 4). A butynamide moiety was incorporated to maintain covalency and 2-pyridyl amide was introduced to the other side of central heterocycle to act as a hydrophobic group. Acalabrutinib was launched in the U.S. in 2017 for the treatment of CLL. It showed potent inhibitory activity in BCR-mediated cell surface expression of CD69 in human peripheral blood mononuclear cells (PBMCs) (EC_50_ = 2.9 nM) and in the human whole blood (hWB) assay (EC_50_ = 9.2 nM). Acalabrutinib showed better selectivity and safety as well as improved off-target effect compared to ibrutinib [55]. Acalabrutinib completed a phase 2 clinical trial in RA in June 2016 (NCT02387762), but the results were not made public. A phase 2, open-label, randomized study of the efficacy and safety of acalabrutinib with best supportive care (BSC) versus BSC alone in patients hospitalized with COVID-19 was completed in November 2020 (NCT04380688). The clinical results demonstrated the effectiveness of acalabrutinib in treating excessive inflammation in the context of severe COVID-19. Off-label use of acalabrutinib for patients with COVID-19 showed normalization of inflammatory markers and decreased oxygen requirements. However, data from the CALAVI phase 2 trials did not meet their primary efficacy endpoints [32].

Tirabrutinib (**8**) (ONO-4059/GS-4059) was initially developed by Ono pharmaceutical to treat B-cell lymphoma and CLL. It was a covalent and potent BTK inhibitor (IC_50_ = 6.8 nM) [48]. In tirabrutinib, a purinone ring was used to function as the heterocyclic core and a butynamide moiety was incorporated to maintain covalent interaction (Figure 4). In 2014, Ono Biomedica licensed the development and commercialization rights of tirabrutinib to Gilead for the treatment of B-cell malignancies and other diseases in all countries except Japan, Taiwan, South Korea, China and the association of Southeast Asian countries. In March 2020, tirabrutinib was approved in Japan for the treatment of recurrent or refractory primary central nervous system lymphoma (PCNSL). In a cell assay using human monocytes stimulated with immobilized hIgG or TLR-9 ligand, tirabrutinib could suppress the production of TNF-α and IL-6. Treatment with tirabrutinib brought about a dose-dependent inhibition of arthritis severity and bone damage in the CIA model [56,57]. Tirabrutinib entered phase 1 clinical trials to evaluate the safety and PK in healthy volunteers and RA patients in 2015 (NCT02626026). Gilead is conducting a phase 2 clinical trial to assess the safety and efficacy of tirabrutinib in adults with active SS (NCT03100942). Ono pharmaceutical is also conducting an open-label, multicenter, phase 2 study that plans to evaluate the efficacy and safety of tirabrutinib in Japanese patients with refractory pemphigus (JapicCTI-184231) [58].

Tolebrutinib (**9**) (PRN2246/SAR442168) was initially developed by Principia BioPharma and was an imidazolonepyridine-based compound (Figure 6). Tolebrutinib was licensed to Sanofi in 2017. It was an irreversible, covalent, oral BTK inhibitor with potent (IC_50_ = 0.7 nM) and durable action, and was designed to access the brain and spinal cord by crossing the blood–brain barrier. Tolebrutinib showed pharmacological inhibition of BTK-dependent diseases in both the CNS and periphery. It showed excellent selectivity for BTK over a number of kinases, except for BLK, BMX, TEC and ERBB4 (IC_50_: 0.6, 1.1, 1.0, and 1.0 nM, respectively). Notably, this BTK inhibitor was demonstrated to provide dose-dependent protection from MS induced in a mouse model of experimental autoimmune encephalomyelitis (EAE) [59]. MS is a chronic inflammatory degenerative disorder, affecting more than 2.3 million people around the world, which results from the gradual destruction of the myelin sheaths that protect nerve cells, and of the nerve cells themselves. B lymphocytes are widely recognized as a critical driver of the disease process. In February 2020, Sanofi announced results for their phase 2b clinical trial with tolebrutinib in relapsing MS (NCT03889639). Specifically, Sanofi announced that tolebrutinib achieved its primary endpoint, significantly reducing disease activity in patients with MS and being well tolerated in the phase 2b trial with no new safety findings. Sanofi initiated four phase 3 clinical trials to evaluate tolebrutinib in the relapsing–remitting (RMS), primary progressive (PPMS) and secondary progressive (SPMS) forms of MS in the middle of 2020 (NCT04410978, NCT04458051, NCT04411641, and NCT04410991).

The replacement of pyrazolo[3,4-d]pyrimidine of ibrutinib with pyrazolo[3,4-d]pyridazinone afforded a novel class of potent irreversible BTK inhibitors and the SAR work resulted in the identification of compound **10** (Figure 6). Compound **10** showed high potency against BTK kinase (IC_50_ = 2.1 nM) and an acceptable PK profile. A docking study of compound **10** with BTK revealed that its binding mode could overlay well with that of ibrutinib. This compound demonstrated significant in vivo efficacy in a mouse CIA model. Of note, the 3 mg/kg regimen of **10** provided equivalent efficacy to ibrutinib at 10 mg/kg. In addition, compound **10** was well tolerated, and no significant body weight loss was observed for different dosages [60].

Olmutinib (**11**) (HM-61713/BI-1482694) was developed by Hanmi Pharmaceutical as an epidermal growth factor receptor (EGFR) tyrosine kinases inhibitor [61] (Figure 6). It has a 2,4-disubstituted thieno-[3,2-d]-pyrimidine scaffold and a typical terminal acrylamide. Olmutinib was first approved in Korea in May 2016 for the treatment of patients with locally advanced or metastatic EGFR T790M-mutation-positive non-small cell lung cancer. Olmutinib could also inhibit BTK, with an IC_50_ of 13.9 nM, and showed potent inhibition against murine lymphocytes proliferation induced by lipopolysaccharide (IC_50_ = 300 nM), which indicated a potential immunosuppressive activity. Adjusting the positions of the two substituents on the central heterocycle, and optimizing the substituents, resulted in the identification of compound **12**. This compound showed equivalent BTK activity (IC_50_ = 29.9 nM) to olmutinib and excellent immunosuppressive activity by inhibiting lymphocytes proliferation (IC_50_ = 284 nM), and also showed very low cytotoxicity. Moreover, compound **12** exhibited negligible activities against EGFR, JAK3, and ITK, indicative of better selectivity than olmutinib [62].

Poseltinib (**13**) (HM-71224/LY-3337641), developed by Hanmi Pharm, is an orally active, irreversible BTK inhibitor (Figure 6). It contained a central 2-amino furopyrimidine moiety and an N-methyl piperazine was used for the hydrophobic group. Poseltinib showed a potent inhibition for BTK, with an IC_50_ of 1.95 nM. It also inhibited other kinases, including BMX, TEC, and TXK, with selectivity ratios of 0.3-, 2.3- and 2.4-fold, respectively. Poseltinib markedly inhibited the phosphorylation of BTK and its downstream messenger molecule PLCγ2 in activated Ramos B lymphoma cells and primary human B cells in a dose-dependent manner. The surface expression of CD69 and CD86 by activated primary human B cells was remarkably inhibited by poseltinib (IC_50_ = 4.2 nM for CD69, and IC_50_ = 7.7 nM for CD86). Furthermore, this compound demonstrated a suppressive effect on the inflammatory cytokines such as TNF-α, IL-6, and IL-1β mediated by FcγR activation. Notably, poseltinib inhibited cytokine production in human plasmacytoid dendritic cells (PDCs) induced by CpG oligodeoxynucleotides (CpG ODN, a TLR9 agonist) [63,64]. It had been demonstrated that the TLR9 pathway is particularly important in SLE [65]. Poseltinib ameliorated experimental arthritis and prevented joint destruction in a mouse CIA model. It significantly reduced erosive bone changes and prevented bone loss. The efficacy of poseltinib at 10 mg/kg and 30 mg/kg was comparable with that of dexamethasone at 0.2 mg/kg. Two lupus models were used to evaluate the therapeutic effect of poseltinib. In MRL/lpr mice, poseltinib effectively inhibited splenic B220^+^GL7^+^, B220^+^CD138^+^, and B220^+^CD69^+^ B cell counts. Furthermore, poseltinib suppressed anti-dsDNA IgG and reduced splenomegaly and lymph node enlargement in both MRL/lpr and NZB/W F1 mice. The compound also prevented skin lesions caused by progression of lupus, and ameliorated renal injury and inflammation in animal models of lupus. In addition, poseltinib improved the survival rate in both lupus models. It is worth noting that there was no mortality during the period of poseltinib administration in both mouse models [63,64]. Poseltinib was licensed to Lilly in 2015. Phase 1 clinical trials were completed by Hanmi for the treatment of RA (NCT01765478). However, phase 2 clinical trials by Lilly to evaluate the drug for the treatment of adult patients with moderate to severe RA were terminated in 2016 due to lack of efficacy (NCT02628028).

One of the strategies of designing targeted covalent inhibitors (TCIs) is to attach a reactive warhead to a reversible lead compound, thus probably affording covalent inhibition with increased potency/selectivity and a prolonged PD effect [66,67]. Replacing the quinazolinone of BMS-935177 with acrylamide conversed the reversible BTK inhibitor into an irreversible one (**14**) with a six-fold increase in potency (IC_50_: 0.46 nM versus 2.6 nM) (Figure 6) [68]. Unfortunately, low plasma concentrations with an aniline metabolite were found following oral dosing of carbazole derivative **14** in a mouse PK study. The aniline metabolite resulted from the acrylamide hydrolysis, constituting the major drug-related compound in circulation. Further structural optimization by scientists at Squibb resulted in the identification of branebrutinib (**15**) (BMS-986195) that possessed a dimethylindole carboxamide core (Figure 6). Introduction of a fluorine atom at C5 of the indole provided a five-fold enhancement in BTK inhibition (IC_50_: 0.1 nM versus 0.52 nM). The X-ray co-crystal structure demonstrated the fluorine atom was involved in a polar interaction with the α-CH of Leu547 as well as a halogen bond with the amide carbonyl of Val546. The indole N1 and the carboxamide made four hydrogen bonds with Thr474, Glu475 and Met477. The 3-amino group on piperidine engaged in a water-mediated hydrogen bond with the backbone carbonyl of Leu547. The co-crystal structure confirmed the key interactions between the inhibitor scaffold and the active site, which endowed branebrutinib with high affinity to the enzyme. The balance between potency and reactivity as well as PK and stability was achieved with the but-2-ynamide warhead paired with the (S)-3-aminopiperadine linker. Branebrutinib provided significantly higher plasma concentrations in a mouse PK study relative to the parent molecule without fluorine (*C*_max_ = 42 μM, AUC = 52 μM·h vs. *C*_max_ = 0.49 μM, AUC = 1.1 μM·h, respectively). Branebrutinib demonstrated a rapid rate of BTK inactivation that was four-fold faster than ibrutinib (*K*_inact_/*K*_i_: 0.97 vs. 0.23). It was highly selective, providing >5000-fold selectivity for BTK over 240 kinases with only the four related Tec family kinases showing less than 5000-fold selectivity (9- to 1000-fold). In B cells stimulated through the BCR, branebrutinib potently inhibited signaling and functional end points, including the calcium flux, production of cytokines, proliferation, and surface CD86 expression. Branebrutinib was also highly effective at inhibiting TNFα production. In the CIA model, once daily oral dosing of branebrutinib led to a dose-dependent inhibition of clinically evident disease, with 21%, 83%, and 92% inhibition observed at the end of the study (38 days duration) at doses of 0.1, 0.5, and 2.5 mg/kg, respectively. Microcomputed tomography of the hind limbs illustrated that branebrutinib provided a dose-dependent protection against pitting, loss of bone mass, woven porous bone, and fusion of the small bones, which was evident in the mice that received only vehicle. In a NZB/W lupus-prone mouse model, this drug provided complete protection from disease-related death over the course of treatment, and dose-dependently inhibited the increase in severe proteinuria. In the in vivo evaluation, branebrutinib showed short plasma T_1/2_ (iv) and T_max_ (po) values across species (0.46−4.3 h and 0.58−1.0 h, respectively), indicating a rapid clearance of the excess inhibitor. The preclinical studies demonstrated that branebrutinib had a very low off-target/nonspecific daily covalent binding burden (<0.10 mg/day) and a desirable safety and tolerability property. In phase 1 studies in healthy subjects, branebrutinib was well tolerated with >99% BTK occupancy (inactivation) at doses of ≥3 mg received once daily (NCT02705989, NCT03245515 and NCT03262740) [69]. Currently, Squibb is also conducting another phase 2 study to assess the safety and effectiveness of branebrutinib treatment in subjects with active SLE or Primary SS, or branebrutinib treatment followed by open-label abatacept treatment in patients with active RA (NCT04186871).

#### 3.1.2. Monoheterocycle (Aminopyrimidine, Pyridinecarboxamide and Pyrazolecarboxamide)

This type of compound features a monoheterocycle core for putative hinge binding. Spebrutinib (**16**) (CC-292/AVL-292) was originally developed by Avila Therapeutics and was acquired by Celgene in March 2012 (Figure 7). It is an orally available, potent, selective and irreversible BTK inhibitor (IC_50_ < 0.5 nM). The compound used 2,4-diaminopyrimidine as the core scaffold. The X-ray co-crystal structure revealed that pyrimidine nitrogen and pendant NH developed double hydrogen bonds to the backbone atoms of hinge Met477. The electronegative fluorine atom was involved in an intramolecular H-bond with the ortho-position NH as well as a weak intermolecular H-bond with the side chain hydroxyl of Thr474. The C-4′ aliphatic ether chain on the aniline at the C-2 position of the pyrimidine core extended to the solvent and the phenolic oxygen was engaged in a water-mediated hydrogen bond with Ala478. The angle between the two aniline planes was about 120° and a weak edge-to-face π-π interaction was formed, with a distance of 3.6 Å. Thus, spebrutinib adopted a “U-shape” conformation [70]. Spebrutinib blocked BCR signaling in the Ramos human Burkitt’s lymphoma cell line through covalent interaction with the Cys481 residue, and selectively inhibited the autophosphorylation of BTK Y-223 and the trans-phosphorylation of its downstream substrates, including PLCγ2 and ERK. It showed high efficacy in CIA model, with 85% and 95% inhibition of disease observed at doses of 10 and 30 mg/kg/day, respectively. In the mouse model, the drug reduced joint and paw swelling and invisible redness of the affected paws in a dose-dependent manner [70,71]. Spebrutinib completed a phase 2 study to evaluate its efficacy and safety in active RA compared to placebo as co-therapy with Methotrexate in July 2017, but the results were not made public (NCT01975610).

Remibrutinib (**17**) (LOU064) (developed by Novartis Pharmaceuticals) contains an aminopyrimidine core and binds covalently to BTK (IC_50_ = 1.3 nM) (Figure 7). The X-ray complex structure revealed that the aminopyrimidine moiety caused double hydrogen bonding interactions with the backbone of hinge residue Met477. The pyrimidine N1 formed a water-mediated hydrogen bond with Lys430 and Thr474. In addition to the covalent binding to Cys481, the carbonyl of the acrylamide formed a hydrogen bond with the backbone NH of Cys481. The fluorophenylcyclopropyl group fit a hydrophobic pocket and stabilized BTK in an inactive conformation. The hydrophobic pocket was established by the side chains of the Gly-rich loop residues Gln412 and Phe413, the catalytic loop residues Asp521 and Asn526, the DFG residue Asp539, as well as Leu542, Ser543, Val546, and Tyr551 from the A loop. It showed potent inhibition toward BCR/CD69 (IC_50_ = 2.5 nM) and FcγR/IL8 (IC_50_ = 18 nM) signaling. The compound also demonstrated excellent best-in-class kinase selectivity from other launched and clinical-stage covalent BTK inhibitors. In the competition binding assay, remibrutinib showed very potent affinity to BTK, with a *K*_d_ of 0.63 nM and with a selectivity of 175-fold against TEC and 857-fold against BMX. Furthermore, the molecule did not show binding to ITK, EGFR, ERBB2, ERBB4, and JAK3 up to 10 μM. Remibrutinib potently inhibited a chronic arthritic inflammation in vivo in a therapeutic treatment setting with a fast onset of action. At a dose of 3 and 10 mg/kg in the rat CIA model, the compound significantly inhibited the bone and cartilage erosion, as well as inflammatory cell infiltration. It also demonstrated potent in vivo target occupancy with an EC_90_ of 1.6 mg/kg after a single oral dose as well as good preclinical tolerability and safety profiles in two species. Administration of the drug in healthy subjects did not raise any safety signals in phase 1 studies (NCT03918980) [72]. Remibrutinib is currently being tested in two phase 2 clinical studies for chronic spontaneous urticaria and SS (NCT04109313 and NCT04035668).

Evobrutinib (**18**) is an orally available, covalent, irreversible BTK inhibitor, developed by Merck, which employs a 4,6-diaminopyrimidine core as the hinge binder (Figure 7). Analysis of the co-crystal structure demonstrated that the aminopyrimidine group established interactions with the gatekeeper residue Thr474 as well as the backbone carbonyl and amide NH of Glu475 and Met477, respectively. The terminal phenyl ring fit in a hydrophobic sub-pocket lined by Val458, Ser538, Asp539, Phe540 and Leu542. Evobrutinib provided excellent potency in the biochemical assay (BTK IC_50_ = 8.9 nM), good solubility and microsomal stability, and acceptable hERG inhibition (Ki = 3.1 μM). In a kinase selectivity assay at 1 μM, only BTK, BMX and TEC were found to show >80% inhibition [73]. Evobrutinib inhibited autophosphorylation of BTK Y223 but not phosphorylation of Y551 in Ramos cells. It also potently inhibited BCR-stimulated B cell activation in PBMCs (CD69: IC_50_ = 15.8 nM) and FcγR signaling activated by a CD64-specific Ab (IC_50_ = 78 nM), as well as the secretion of cytokines IL-2, TNF-a, IL-17A, and IL-6. This inhibition translated into efficacy in animal models for the B cell-driven autoimmune diseases RA and SLE. In the CIA rat model, ankle-swelling was significantly reduced at doses of 3, 10, and 30 mg/kg. In the SLE-prone NZB/W F1 mouse model, evobrutinib prevented proteinuria and reduced histological kidney damage. In addition, features of SLE such as dysregulated cholesterol and hemoglobin levels were normalized. This is the first report describing these effects of a BTK inhibitor in an animal model for SLE [74]. A phase 2b study of evobrutinib in patients with RA was completed in September 2020, but no results were disclosed (NCT03233230). Merck is also conducting two phase 3 clinical studies using this drug for the treatment of subjects with relapsing MS (NCT04338061 and NCT04338022).

Orelabrutinib (**19**) (ICP-022), developed by InnoCare Pharma, features a pyridinecarboxamide core (Figure 7). It is an orally active, covalent, irreversible BTK inhibitor with high potency (BTK IC_50_ = 1.6 nM) and was approved in China for MCL, CLL and small lymphocytic lymphoma (SLL) in December 2020. In a screening assay against 456 kinases at 1 μM, orelabrutinib only targeted BTK with >90% inhibition, indicating its superior kinase selectivity. Orelabrutinib demonstrated excellent PK/PD profiles that distinguished it from other BTK inhibitors. Sustained ~100% BTK occupancy at 24 h was achieved with once daily dosing regimens of 50 mg and above. Orelabrutinib was first launched in China in December 2020 for the treatment of CLL, MCL and SLL [75]. Currently, a phase 2 study of orelabrutinib is being carried out for patients with relapsing–remitting MS (NCT04711148). InnoCare Pharma is also conducting a phase 1/2 trial using orelabrutinib for the treatment of patients with SLE (NCT04305197).

BI-BTK-1 (**20**) was reported to be a potent, selective, small molecule inhibitor of BTK (Figure 7) that can form an irreversible covalent bond between the electrophile present in R’ moiety and cysteine 481, but its molecular structure has not yet been fully disclosed. The pyrazole carboxamide is believed to form H-bond interactions with the hinge region of BTK. BI-BTK-1 potently inhibited BTK enzymatic activity and BCR-stimulated B cell activation (BTK IC_50_ = 0.9 nM; CD69 IC_50_ = 2.4 nM), as well as the secretion of cytokines from immune complex-stimulated human monocytes. BI-BTK-1 was evaluated in an inducible model (NTN, nephrotoxic nephritis) of Lupus nephritis (LN) in which mice received nephrotoxic serum (NTS) containing anti-glomerular antibodies. In the NTN model, BI-BTK-1 ameliorated nephritis and prevented renal damage in a dose-dependent manner. The inhibition of BTK by BI-BTK-1 significantly reduced the levels of LN-relevant inflammatory cytokines and chemokines. Notably, BI-BTK-1 reversed established proteinuria and improved renal histopathology in therapeutic NTN mice [76]. In two spontaneous LN models, the NZB/W mouse and the MRL/lpr mouse, BI-BTK-1 treatment attenuated kidney disease and significantly increased survival. In addition, BI-BTK-1 decreased kidney immune cell infiltration and diminished the local expression of nephritis-associated inflammatory mediators in the kidney. Importantly, BI-BTK-1 reversed established kidney disease in MRL/lpr mice [25]. In another murine study using the MRL/lpr mouse model, treatment with BI-BTK-1 remarkably ameliorated the lupus-associated cutaneous disease phenotypes. Specifically, BI-BTK-1-treated mice had macroscopically and histologically improved skin lesions, reduced cutaneous cellular infiltration, and diminished expression of inflammatory cytokine as compared with the control mice. MRL/lpr mice also spontaneously exhibited a neuropsychiatric disease phenotype, including cognitive dysfunction and depression-like behavior. In the MRL/lpr mouse model, BTK inhibition markedly improved cognitive function, and reduced the accumulation of T cells, B cells, and macrophages in the choroid plexus [26].

#### 3.1.3. Fused Tricyclic Heterocycle (Imidazoquinoxaline)

In 2011, Pfizer developed a series of irreversible BTK inhibitors based on imidazoquinoxaline (a fused tricyclic scaffold), and compound **21** was selected for detailed evaluation (Figure 7). Compound **21** was a potent BTK inhibitor, with an IC_50_ of 1.93 nM, and effectively inhibited the in vitro proliferation of human B cells following stimulation through BCR with anti-IgM. The introduction of fluoro substituent at C-7 position retained good BTK inhibitory activity and improved selectivity over LYN inhibition relative to the C-7 unsubstituted parent. The authors carried out a crystallographic study of this compound with a gate-keeper variant (Phe435Thr) of ITK whose ATP-site shares 70% sequence identity with BTK. The crystal structure illustrated that Cys442 bound covalently to the butenamide. The imidazo nitrogen (N2) was involved in a hydrogen bond with the backbone NH of hinge residue Met438, while the C4-amino group formed another hydrogen bond with the side chain hydroxy of gatekeeper residue Thr435. The inhibition of BTK autophosphorylation, by compound **21**, at Y223 in Ramos cells was observed (IC_50_ = 48 nM). Compound **21** exhibited favorable pharmacokinetic properties with a t_1/2_ of 1.5 h (iv dose), an AUC of 876 h·ng/mL (po dose), and an oral bioavailability of 34%. In a semi-therapeutic CIA model, treatment with compound **21** significantly inhibited the progression of the disease relative to the vehicle control at 10 and 3 mg/kg from day 7 and 5, respectively [77].

### 3.2. Covalent Reversible Inhibitors

The irreversible acrylamide- or alkynyl amide-based kinase inhibitors have been shown to form permanent covalent adducts with cysteine-containing kinase and non-kinase off-target proteins. These irreversible adducts have always been a concern in drug discovery owing to the relationship between covalent drug binding and the potential for idiosyncratic adverse drug reactions [52,53]. Therefore, a reversible covalent inhibitor might provide reduced risk of idiosyncratic drug toxicity and be safer than an irreversible one.

**Figure 8:**
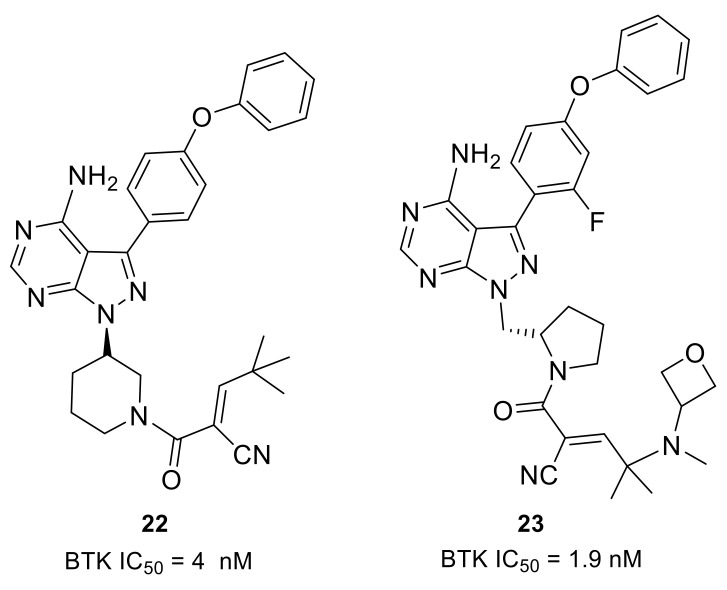
The representative structures of covalent reversible inhibitors.

Scientists from Principia Biopharma explored the possibility of engaging BTK Cys481 with reversible covalent inhibitors. By using the cysteine-reactive cyanoacrylamide electrophile, they identified potent and selective BTK inhibitors that demonstrated biochemical residence times spanning from minutes to 7 days [46]. Compound **22** potently inhibited the BTK enzyme (IC_50_ = 4 nM) and showed sustained BTK occupancy in both cellular and biochemical assays (Figure 8). However, its low aqueous solubility and poor oral bioavailablility limited further development. Structural modifications on the linker and the terminal capping group were enabled in compounds **23** and **3** (rilzabrutinib) (Figure 4 and Figure 8). In compound **23**, the (*S*)-methylpyrrolidine was used as a linker and an amino-oxetane was attached as the capping group. Compound **23** was a potent BTK inhibitor (IC_50_ = 1.9 nM) and showed high selectivity in a 254-kinase panel assay. With a residence time of 167 h, cyanoacrylamide **23** was among the most durable reversible inhibitors so far identified. Furthermore, compound **23** showed reversible covalent binding, as demonstrated by its rapid and quantitative dissociation upon BTK proteolysis. Compound **23** was demonstrated to have good aqueous solubility (126 μM at pH 7), be orally bioavailable and provide sustained BTK engagement in vivo.

Another oral reversible covalent inhibitor, rilzabrutinib (**3**), was very potent against BTK (IC_50_ = 1.3 nM). The compound showed high selectivity when tested against a panel of 251 other kinases, except for BMX, TEC, RLK and BLK (IC_50_: 1.0, 0.8, 1.2, and 6.3 nM, respectively). Cysteine targeting of BTK by rilzabrutinib led to a slow off-rate, demonstrated by a retention of 79 ± 2% of the binding to BTK in PBMC 18 h after washing away the molecule in vitro. The covalent binding that targeted cysteine was completely reversible after target denaturation. Rilzabrutinib inhibited potently anti-IgM-induced human B cell proliferation (IC_50_ = 5 nM) and B cell CD69 expression (IC_50_ = 123 nM) and did not show cytotoxicity in the epithelial cell line HCT-116. In vivo rilzabrutinib showed enduring pharmacodynamic effects after the compound had been cleared from circulation. Additionally, rilzabrutinib dose-dependently reversed and completely inhibited CIA in rats [78,79]. Clinical trials indicated that rilzabrutinib was safe and well-tolerated following oral administration, and achieved high, sustained levels of BTK occupancy in PBMCs [80]. Immune thrombocytopenia (ITP) is a rare autoimmune disease that causes high risk of bleeding, excessive bruising, fatigue and potential for life-threatening intracranial bleeding due to the destruction of platelets. Rilzabrutinib was designated as an orphan drug for the treatment of ITP in the U.S. in October 2018. Currently, rilzabrutinib is recruiting adults and adolescents with persistent or chronic ITP for a phase 3 clinical trial (NCT04562766). Pemphigus refers to a group of rare, debilitating, autoimmune diseases that cause blistering of the skin and mucous membranes. Rilzabrutinib was also designated as an orphan drug for the treatment of pemphigus vulgaris in the U.S. in June 2017. A phase 3 study to evaluate rilzabrutinib in patients with pemphigus is currently ongoing (NCT03762265). Principia Biopharma is also conducting a phase 2 clinical program for rilzabrutinib that aimed at patients with a rheumatologic indication, IgG4-related disease (RD), which is driven by chronic inflammation, immune cell infiltration and fibrosis within organs that, if left untreated, can lead to severe morbidity including organ dysfunction and organ failure (NCT04520451).

### 3.3. Non-Covalent Reversible Inhibitors

In this section, the inhibitors of each type are further categorized based on their core heterocycles.

#### 3.3.1. Fused Bicyclic Heterocycle (Imidazopyrazine, Pyrrolopyrimidine, Cinnoline and Quinoline)

**Figure 9:**
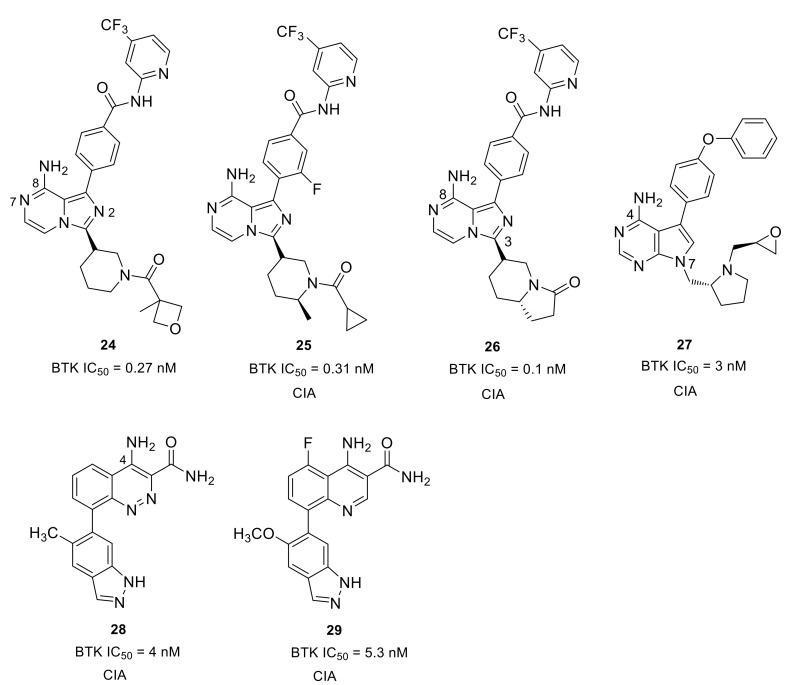
The representative structures of non-covalent reversible BTK inhibitors with fused bicyclic heterocycle scaffolds and their progress in the treatment of autoimmune and inflammatory diseases.

Merck developed a series of non-covalent, reversible and selective BTK inhibitors based on an 8-amino-imidazo[1,5-a]pyrazine core. Compounds **24** and **25** displayed potent BTK inhibitory activity with IC_50_ values of 0.27 nM and 0.31 nM, respectively (Figure 9). These two compounds had excellent kinase selectivity. For the kinases from the Tec and Src family, the selectivity ratios over the BTK enzyme were greater than 50-fold, except that compound **25** was 34-fold for BMX and 38-fold for LCK. In the human PBMC functional assay and whole blood assay, compound **25** demonstrated better activity when compared to **24** (IC_50_: 4.0 nM vs. 8 nM; 94 nM vs. 120 nM). The co-crystal of compound **24** with BTK enzyme showed that the 2-aminopyridine formed a bidentate hydrogen bond interaction with the side chain hydroxyl of Ser538 and the amide nitrogen of Asp539. The pyridine nitrogen was also involved in water-mediated bridging interactions with Ser538 and Val458. The trifluoromethylpyridine moiety fit in the hydrophobic H3 pocket. The 8-amino and N7 on the imidazo[1,5-a]pyrazine each formed one hydrogen bond with the hinge residues Glu475 and Met477, respectively, and the 8-amine group also engaged in a H-bond to the side-chain alcohol of gatekeeper Thr474, while the N2 atom was involved in a water-mediated hydrogen bond with the backbone NH of Lys430. The amide carbonyl on the piperidine formed a hydrogen bond with the backbone NH of Cys481. Compound **25** showed a different binding mode around the trifluoromethylpyridine relative to compound **24**. There were no bidentate hydrogen bond interactions with Ser538 and Asp539 and the trifluoromethylpyridine moiety extended more deeply into the pocket. In the BTK-compound **25** complex, the cyclopropylamide substituent on the piperidine had a trans conformation forced by the methyl group, with the carbonyl oxygen forming a hydrogen bond with the C481 NH [81]. The Merck scientists succeeded in cyclization of the α-carbon of the amide with the piperidine 2-carbon to afford a bicyclic ring substituent in the 3-position (Figure 9). The ring constraint locked the carbonyl in the required direction to form a hydrogen bond with backbone amide NH of C481, which was demonstrated in the X-ray crystal structure of a bicyclic analog bound to BTK. When compared to **25**, compound **26** showed improved potency in the enzymatic, cellular and whole blood assays (IC_50_: 0.1 nM, 2.5 nM, and 24 nM, respectively), as well as better selectivity against Tec and Src family kinases. Furthermore, the lactam constrained compound **26** displayed better PK profile in rats with low clearance, long half-lives and high oral bioavailability relative to **25**. In a prophylactic rat model of CIA, both **25** and **26** displayed dose-dependent efficacy (1, 3, 10, and 30 mg/kg) in reducing the paw thickness [82].

To develop a non-covalent reversible BTK inhibitor, Chen’s group tried to replace the acryloyl group in compound **6** (an irreversible inhibitor) with some functional groups that cannot work as Michael addition receptors. After exploration of the SAR, the (R)-pyrrolidin-2-yl-methyl group with a (S)-epoxy substituent was introduced at the 7-position of the pyrrolo[2,3-d]pyrimidine core, and the benzo[d][1,3]dioxol-5-yl moiety was replaced by 4-phenoxyphenyl group, thus affording compound **27**, which combines the best structural fragments (Figure 9). Compound **27** exhibited potent activity (IC_50_ = 3.0 nM) against BTK and inhibited BTK Y223 auto-phosphorylation and PLCγ2 Tyr1217 phosphorylation. Compound **27** had low inhibition to HER4, EGFR, HER2, JAK3 that share a cysteine residue at structurally identical positions. For other Tec family kinases (TEC, BMX, ITK, and TXK), the selectivity ratios over the BTK enzyme were between 15 and >333-fold. However, 3-fold unsatisfactory selectivity over BLK and LYN in SRC family kinases was observed. In a mice model of CIA, this compound exhibited dose-dependently reduced clinical scores, inhibited joint inflammation and significantly reduced paw thickness. Although compound **27** had a slightly lower clinical score than ibrutinib in the CIA model, the development of compound **27** and its analogs provided new insights towards the discovery of reversible BTK inhibitors as novel anti-arthritic agents [83].

Scientists at Takeda Pharmaceutical developed an orally available small molecular BTK inhibitor **28** (BTK IC_50_ = 4.0 nM) containing a cinnoline carboxamide scaffold (Figure 9). X-ray co-crystal structure analysis revealed that the 4-amino and 3-carboxamide on the cinnoline ring caused three H-bond interactions with the BTK hinges Glu475 and Met477, respectively. The NH_2_ of 3-carboxamide formed a direct hydrogen bond with the side chain of gatekeeper Thr474 and simultaneously caused water-mediated bridging interactions with Thr474 and Ser538. The two nitrogens of cinnoline were involved in hydrogen bonding interactions with Lys430 and Asp539, which were mediated by a water molecule. The two nitrogen atoms of indazole made two H-bonds with Gly414 and Phe413 of the P-loop, respectively. The selectivity ratios of **28** over the Tec and Src family of kinases were between 18- and 160-fold, respectively. However, the compound displayed <10-fold selectivity ratios against ALK2, LIMK1, and TNK2. Although compound **28** reduced paw swelling in a dose-dependent manner in a rat CIA model, the poor aqueous solubility (<0.05 μg/mL at pH 7.4) limited its in vivo study [84]. Given the stability and inhibitory activity of the compound 28, Rao’s group carried out an optimization on it and discovered a 4-aminoquinoline-3-carboxamide derivative **29**, which showed significantly improved drug-like properties, especially in terms of aqueous solubility (Figure 9). Compound **29** exhibited over ~150-fold improvement in aqueous solubility (7.3 μg/mL at pH 7.4) compared with cinnoline **28**. The quinoline derivative **29** showed a potent inhibitory effect on both BTK-WT (IC_50_ = 5.3 nM) and BTK-C481S (IC_50_ = 39 nM), as well as dose-dependent and efficient inhibition of BTK Tyr223 autophosphorylation. Moreover, compound **29** was more selective for BTK than other members of the Tec and SRC kinase families. The selectivity ratios were all greater than 30-fold, except for BLK (28-fold). In the mouse CIA model, compound **29** efficiently reduced paw swelling without a loss in body weight, implying that this inhibitor was both efficacious and well tolerated [85].

#### 3.3.2. Monoheterocycle (Aminopyrazinone, Aminopyridazinone, Aminopyridinone, Aminopyrimidine, and Amino-1,3,5-triazine)

CGI Pharmaceuticals identified CGI-1746 (**2**) as a potent and reversible BTK inhibitor (IC_50_ = 1.9 nM) that features a pyrazinone core (Figure 4). CGI-1746 inhibited both transphosphorylation (Tyr551) and autophosphorylation (Tyr223) dose-dependently and efficiently, as well as anti-IgM-induced human B cell proliferation (IC_50_ = 42 nM). It also blocked FcγR-induced signaling and inflammatory cytokine production in murine macrophages. The SAR studies revealed that the t-butylphenyl group and the para-amide were both required for potent binding, while changing the size of the acyl moiety dramatically decreased the activity. CGI-1746 was specific for BTK, with ~1000-fold selectivity over Tec and Src family kinases. The excellent potency and selectivity of CGI-1746 could be ascribed to its optimal molecular structure, which can simultaneously form favorable interactions with both the hinge region and the H3 pocket of BTK, as mentioned above. In the CIA model, CGI-1746 prevented arthritis and significantly reduced autoantibody levels. CGI-1746 (100 mg/kg bid) also demonstrated efficacy in preventing the development of arthritis in the CAIA model [28]. However, the poor ADME properties hindered its further development (clearance (CL) = 87 mL/min/kg; oral bioavailability (F%) < 5%).

**Figure 10:**
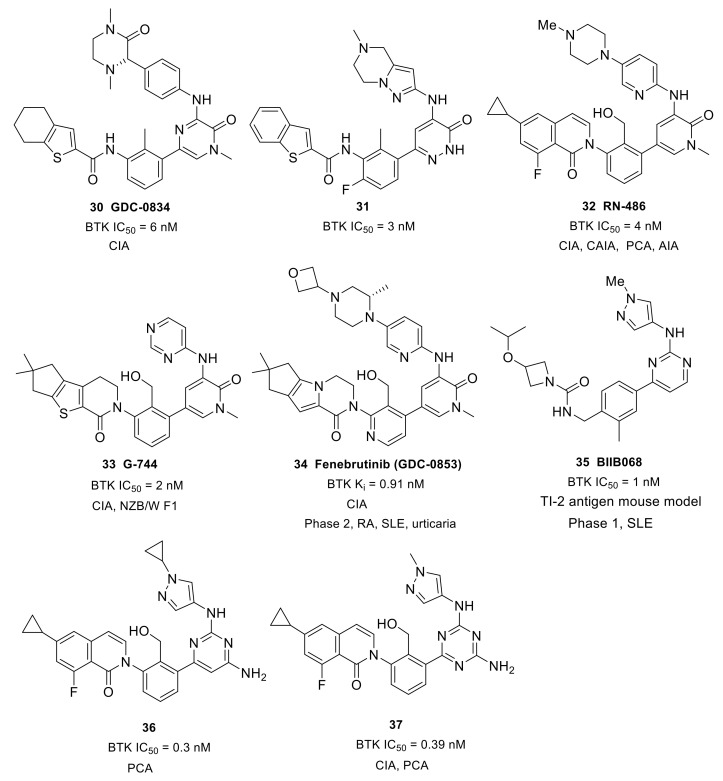
The representative structures of non-covalent reversible BTK inhibitors with monocyclic heterocycle scaffolds and their progress in the treatment of autoimmune and inflammatory diseases. PCA, passive cutaneous anaphylaxis; AIA, Adjuvant-Induced Arthritis.

With the purpose of developing an orally bioavailable small-molecule inhibitor for RA, Genentech and Gilead (Gilead acquired CGI Pharm. in 2010) carried out systematic structural modification on CGI-1746 around the central three cycles in the scaffold. Tetrahydrobenzothiophene was identified as the optimal alternative to the para-*t*-butylphenyl group after screening over 100 analogs. While maintaining the tetrahydrobenzothiophene moiety, replacing the morpholine–amide section with (R)-dimethyl-3-oxopiperazine afforded the clinical candidate GDC-0834 (**30**) with improved PK profiles (CL = 4.4 mL/min/kg; F = 35%) (Figure 10). GDC-0834 inhibited BTK in vitro with an IC_50_ of 6 nM, and showed an EC_50_ of 60 nM in a cell-based CD86 assay. In human whole blood, GDC-0834 inhibited anti-IgE-stimulated CD63 expression with an EC50 of 0.35 μM, and anti-IgD stimulated CD69 expression with an EC50 of 0.38 μM. Moreover, GDC-0834 maintained a similar kinase selectivity profile as CG1-1746, with the exception of BMX (3-fold greater). The crystal structure of GDC-0834 in BTK confirmed a near-identical binding mode to CGI-1746. GDC-0834 demonstrated efficacy in a rat CIA model, inducing a decrease in ankle swelling and a reduction in morphologic pathology in a dose-dependent manner. Unfortunately, in a single dose phase 1 trial in healthy volunteers, GDC-0834 was found to be highly labile at the exo-cyclic amide bond that linked the tetrahydrobenzothiophene to the aniline, resulting in a suspension of clinical trials [86]. This cleavage, in metabolic processes, was demonstrated to result from a non-CYP mediated pathway that was more prevalent in human than preclinical species such as mice, rats, dogs, and monkeys. Subsequent efforts were focused on reducing metabolic cleavage of this amide, and resulted in the identification of pyridazinone as the preferred hinge-binding motif. Further optimization around the central phenyl-pyridazinone generated a potent inhibitor **31** (IC_50_ = 3 nM) with moderate stability in human liver microsomes and low clearance in human hepatocytes (Figure 10). Compound **31** showed excellent kinase selectivity and had no significant off-target activity. Compound **31** demonstrated potent inhibition of anti-IgE-stimulated CD63 and CD86 expression, as well as anti-IgM-stimulated B-cell proliferation, in murine B cells, with EC_50_ values of 257 nM, 91 nM, and 6 nM, respectively. In addition, compound **31** strongly inhibited immune-complex triggered cytokine production by human monocytes (EC_50_ = 37 nM (TNFα); EC_50_ = 22 nM (IL1β)). In a comparison of the co-crystal structures of the inhibitor in complex with BTK, compound **31** had a nearly identical binding configuration to GDC-0834 [87].

Roche Inc. developed the pyridinone-based potent BTK inhibitor RN-486 (**32**) (IC_50_ = 4.0 nM) (Figure 10). As compared with CGI-1746, the pyrazinone core was replaced by a pyridinone ring, and the central toluene was replaced by benzylic alcohol. The co-crystal structure of the inhibitor–BTK complex showed that the alcohol simultaneously formed hydrogen bonding interactions with K430 and D539 and the central phenyl ring occupied the H1 pocket, which significantly boosted ligand affinity, thus resulting in potent inhibition. The carbonyl on isoquinolone formed a direct hydrogen bond with Lys430 and a water-mediated hydrogen bond with both Phe413 and Gly414. Fluorine substitution on the benzene ring that was ortho to the carbonyl group led to an approximate 10-fold increase in potency. The co-crystal structure also demonstrated that the strongly electronegative fluorine was within the van der Waals distance to the primary amine of K430 and the aromatic hydrogen at the ortho position of F413. The cyclopropyl group on isoquinolone was directed to the bottom of the H3 pocket. The compound showed excellent selectivity when tested against a panel of 369 kinases, and favorable PK profiles in rat and mouse models. RN-486 potently inhibited anti-IgM-induced phosphorylation of Btk (Tyr551) and PLCγ2 (Tyr1217) in isolated B cells. RN486 also potently inhibited IgG-FcγR-induced TNFα production in monocytes and the IgE-FcεR-mediated release of histamine in mast cells, with IC_50_ values of 7.0 nM and 2.9 nM, respectively. The results indicated the potential of RN486 in the inhibition of hypersensitivity responses mediated by BCR and FcR. In the passive cutaneous anaphylaxis (PCA) model induced by anti-OVA IgE, RN486 markedly inhibited severe cutaneous hypersensitivity responses, such as skin wheals and dye extravasation around the sites of injection. In the CIA model, RN486 suppressed arthritis in a dose-dependent manner and inhibited ex vivo IgD-stimulated CD69 expression in blood B220^+^ cells at 3 and 6 h post-dose at all doses. In the CAIA model, the compound completely prevented anticollagen antibody-induced arthritis. RN486 inhibited inflammation and bone erosions in an Adjuvant-Induced Arthritis (AIA) model either alone or in combination with Methotrexate [88,89].

In order to overcome the shortcoming of rapid amide bond hydrolysis in humans in GDC-0834 and its analogs, Genentech used an aminopyridinone core and a tricycle amide in the solvent-exposed region, which generated more potent inhibitors than the uncyclized precursors. Further incorporation of a hydroxy at the methyl group on the central benzene improved BTK binding potency. The resulting compound, G-744 (**33**), had a gem-dimethyl substituted 6-5-5 tricyclic ring system (Figure 10). The comparison of co-crystal structures revealed that G-744 had a nearly identical binding mode to RN-486. G744 displayed excellent BTK biochemical and cell potency (BTK IC_50_ = 2 nM; CD86 IC_50_ = 64 nM). In human whole blood, G-744 showed potent inhibition of BCR-stimulated CD69 expression in B cells (EC_50_ = 87 nM). G-744 also inhibited BCR-stimulated B-cell proliferation in human B-cells (EC_50_ = 22 nM) and production of the inflammatory cytokine TNFα in human monocytes (EC_50_ = 33 nM). In a screening assay against 285 kinases, G-744 showed >1000-fold BTK selectivity against all kinases tested except for EphA7 (428-fold) and Fgr (868-fold). The superb kinase selectivity of G-744 made it suitable as a tool molecule to probe the biology of BTK, as the results would not be influenced by off-target activity. In the rat CIA model, oral dosing with the drug at 6.25, 12.5, and 25 mg/kg b.i.d. induced inhibition of BTK Y223 phosphorylation in whole blood and significant dose-dependent inhibition of ankle thickness [90]. Moreover, G-744 was efficacious, and superior to BAFF blockage or SYK inhibition, in the abrogation of severe lupus nephritis in both spontaneous and IFNα-accelerated lupus in NZB/W F1 mice [43].

While maintaining the core structure of G-744, Genentech developed another tricyclic, potent, non-covalent BTK inhibitor—fenebrutinib (**34**) (GDC-0853) (Figure 10). The co-crystal structure revealed that the pyridone carbonyl and pendant NH formed three hydrogen bonds with the backbone atoms of hinge Met477; simultaneously, the weak polar interactions between the N-methyl and backbone carbonyl of Glu475 and the side chain hydroxyl of gatekeeper Thr474 were observed. The hydoxymethyl on the pyridine formed two hydrogen bonds with Lys430 and Asp539 as well as a water-mediated hydrogen bond with Ser538. The pyridyl ring occupied the shallow H1 pocket established by Leu408, Gly409, Thr410 and Val416. The fused tricyclic ring fragment fit well in the deep H3 pocket, similarly to the t-butylphenyl in CGI-1746, and stabilized BTK in an inactive conformation. The gem-dimethyl cyclopentane was directed to the bottom of the H3 pocket formed by Asp521, Ser543 and Tyr551. The terminal oxetanylpiperazine motif extended into the solvent. SAR work demonstrated that introducing the 2-(S)-methyl substituent on the piperazine resulted in the best overall profile. Fenebrutinib had a BTK enzyme Ki of 0.91 nM and a BCR-induced CD69 IC_50_ of 8 nM in human whole blood. It also potently inhibited anti-IgM-induced BTK Tyr223 phosphorylation (IC_50_ = 3.1 nM) and anti-IgM-induced B-cell proliferation (IC_50_ = 1.2 nM), as well as FcγR-dependent TNFα production in human monocytes (IC_50_ = 1.3 nM). Furthermore, in in vitro transfection tests, GDC-0853 blocked the cellular Y223 autophosphorylation of WT Btk and the C481S mutant. When compared with six other clinical stage BTK inhibitors in kinase screening assays, fenebrutinib was found to be the most selective compound for BTK and inhibited only 3 of the 286 off-target kinases. Biochemical kinase assays showed that fenebrutinib displayed > 130-fold selectivity ratios against these three off-target kinases (BMX, Fgr, and SRC). In the development of the CIA model, fenebrutinib dose-dependently reduced ankle thickness following qd and bid dosing regimens. In phase 1 studies of autoimmune diseases in healthy volunteers, fenebrutinib was very well tolerated with no severe adverse events, no safety signals and no dose-limiting toxicities [91]. Genentech completed a phase 2 clinical study of the safety and efficacy of fenebrutinib in participants with moderate to severe active SLE in July 2019, which failed due to lack of efficacy (NCT02908100). Two phase 2 trials were conducted to evaluate the safety and efficacy of fenebrutinib in patients with RA (NCT02833350 and NCT02983227). For patients with RA and an inadequate response to methotrexate (MTX), greater efficacy was achieved with fenebrutinib (150 mg once daily or 200 mg twice daily) compared to placebo, and response rates with fenebrutinib were numerically similar to those observed with the TNF inhibitor adalimumab. In addition, fenebrutinib demonstrated effectiveness in patients who had diseases that were refractory to therapies beyond MTX. The clinical evidence supported the rationale of targeting BTK by fenebrutinib in two different patient populations with active RA [92]. These phase 2 trials were completed in July 2018 and in July 2019, respectively, but no further clinical trials have been ongoing since then. In October 2019, Genentech completed a phase 2 clinical trial to evaluate the efficacy, safety and pharmacokinetics of fenebrutinib in participants with refractory chronic spontaneous urticaria, but no results were disclosed (NCT03137069).

Scientists at Biogen developed a potent, reversible BTK inhibitor BIIB068 (**35**) that had good overall drug-like properties for oral dosing and good ADME properties (Figure 10). BIIB068 featured a 2-aminopyrimidine core and exhibited potent biochemical and cellular activity (BTK IC_50_ = 1 nM, human whole blood pBTK IC_50_ = 0.12 μM). In a screening assay over 395 kinases, only nine kinasese exhibited >70% affinity toward BIIB068 relative to control at 1 μM in addition to BTK. Further affinity measurement demonstrated that BIIB068 showed >400-fold selectivity for BTK versus all nine kinases, including the structurally similar Tec family members (BMX, ITK, TEC and TXK). The X-ray co-crystal structure of BIIB068 bound to BTK revealed that the 2-aminopyridimine motif acted as a dual hydrogen-bond acceptor/donor with hinge Met477. The methyl group on the phenyl ring was positioned under the Gly-rich P-loop. The urea carbonyl engaged in a direct hydrogen bond with Lys430 and was also involved in hydrogen bonding interactions with Phe413 and Gly414 mediated by a water molecule. The benzyl urea linker oriented the azetidine moiety into the H3 pocket, which sequestered Tyr551 in an inactive conformation. To understand the in vivo efficacy, BIIB068 was tested in a thymus-independent type 2 (TI-2) antigen mouse model that had previously been reported to be BTK dependent. Dose-dependent reductions in antigen-specific antibody were observed in mice treated BID with BIIB068. In phase 1 studies in SLE completed in March 2017 (NCT02829541), BIIB068 was well tolerated and effective at inhibiting BTK phosphorylation in healthy volunteers. BIIB068 also inhibited B-cell activation, though the inhibition was not as robust as inhibition of BTK phosphorylation. However, no further clinical trials have been ongoing since 2017 [93].

A 2,4-diaminopyrimidine derivative **36** was developed by Carna Biosciences, Inc. (Figure 10). Compound **36** potently inhibited BTK (IC_50_ = 0.3 nM) and anti-IgM-induced phosphorylation of BTK Tyr223 in cells. Moreover, compound **36** exhibited a high selectivity profile in a kinase-screening assay in which the compound only inhibited two kinases within the panel (BMX and TEC, 70% and 92% inhibitions at 0.3 μM concentration, respectively). A docking model between BTK and **36** was built to analyze the intermolecular interactions. The pyrimidine N1 and 2-amino group made bidentate hydrogen bonds with the backbone amide of Met477 in the hinge. Additionally, the 6-NH_2_ group on pyrimidine was positioned within the hydrogen bond distance with the side chain of Thr474 and the backbone carbonyl of Glu475. The cyclopropyl group on the isoquinolone moiety occupied the large hydrophobic H3 pocket, while the cyclopropyl group at the pyrazole ring was exposed to the solvent region. Oral administration of compound **36** at 30 mg/kg significantly inhibited the PCA reaction of mice with an inhibition rate of 46% as compared to vehicle control. Unfortunately, compound **36** was found to inhibit hERG channel, which impeded the further development of this compound [94]. To eliminate the inhibitory activity of hERG, scientists at Carna turned their attention to replacing the 2,4-diaminopyrimidine core in **36** with a N-containing heteroaryl group, which led to the discovery of a series of 2,4-diamino-1,3,5-triazine derivatives. Structural optimization afforded a potent, selective, reversible BTK inhibitor **37**. Replacement of the pyrimidine core with a 1,3,5-triazine ring retained potency (BTK IC_50_ = 0.39 nM) and provided a 2-fold increase in aqueous solubility (20 μM vs. 9.8 μM) compared to compound **36**. As expected, inhibitor **37** demonstrated reduced hERG inhibition (IC_50_ = 24 μM) relative to compound **36** (IC_50_ = 14 μM). Furthermore, the triazine derivative showed excellent kinase selectivity in screening assay; only two Tec-family kinases were inhibited at 0.3 μM (BMX and TEC, 80% and 99.5% inhibition, respectively). Compound **37** potently inhibited the phosphorylations of Tyr551, Tyr223, and PLCγ2, with IC_50_ values of 19, 14, and 26 nM, respectively, and significantly inhibited anti-IgM-induced B-cell activation in human PBMC, with an IC_50_ of 13 nM. In a PCA model in mice, oral administration of **37** at 30 mg/kg markedly inhibited the PCA reaction, and its inhibition rate was 69% when compared to that of the vehicle control. In a mouse CIA model, compound **37** markedly reduced paw swelling and joint inflammation in a dose-dependent manner [95].

#### 3.3.3. Fused Tricyclic Heterocycle

**Figure 11:**
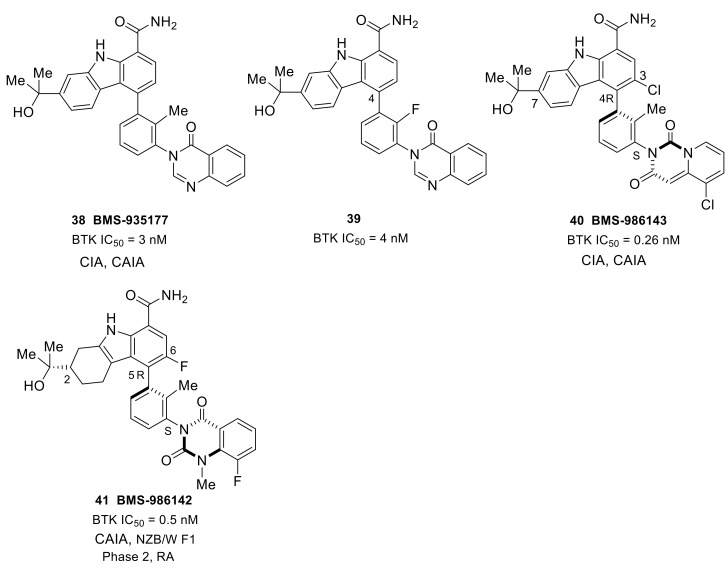
The representative structures of non-covalent reversible BTK inhibitors with fused tricyclic heterocycle scaffolds and their progress in the treatment of autoimmune and inflammatory diseases.

Bristol Myers Squibb developed the orally available, reversible BTK inhibitor BMS-935177 (**38**) with an IC_50_ of 3 nM based on the carbazole scaffold (Figure 11). It demonstrated good kinase selectivity when tested against a screening panel of 384 kinases. Biochemical kinase assays showed the compound to be more potent against BTK than the other Tec family kinases (TEC, BMX, ITK, and TXK) over which the drug was between 5- and 67-fold more selective. Replacement of the C4 toluene linker with a fluorobenzene provided compound **39** with similar BTK inhibitory activity and lowered the PK properties relative to BMS-935177. BMS-935177 exhibited a dose-dependent reduction with once daily oral doses of 10, 20 and 30 mg/kg in a mouse CIA model. In the B cell independent mouse CAIA model, BMS-935177 was also efficacious [96]. Although BMS-935177 showed desirable efficacy in rodent models of RA, undesired side effects were found during tolerability studies, which could be attributed to the existence of a mixture of four interconverting atropisomers. Isolation of a single, rotationally stable atropisomer, BMS-986143 (**40**), was achieved by replacing the quinazolinone in BMS-935177 with a quinazolinedione to lock the lower atropisomeric axis, and by introducing small substituents at C3 to lock the carbazole C4 atropisomeric axis. BMS-986143 provided an ~11-fold enhancement of BTK inhibition (IC_50_ = 0.26 vs. 3 nM), and a 6-fold increase in human whole blood potency (IC_50_ = 90 vs. 550 nM) when compared to BMS-935177, as well as improved kinase selectivity. BMS-986143 exhibited dose-dependent inhibition of clinical disease progression in both CIA and CAIA models [97].

BMS-986142 (**41**) is a single, rotationally stable atropisomer that incorporated a tetrahydrocarbazole core (Figure 11). Among the tetrahydrocarbazole analogs, the fluoro-substituted compound **41** provided the most desirable activity (BTK IC_50_ = 0.5 nM, Ramos IC_50_ = 9 nM, and hWB IC_50_ = 90 nM), along with an acceptable PK profile and a clean liability profile in mice. In B cells stimulated through BCR, BMS-986142 potently inhibited calcium flux, production of cytokines, proliferation, and surface CD86 expression. In addition, BMS-986142 was very effective in inhibiting TNFα production in human PMBC cells (IC_50_ = 3 nM). BMS-986142 demonstrated excellent kinase selectivity in an assay against 384 kinases, and only five kinases were inhibited with less than 100-fold selectivity for BTK. Out of those, four were Tec family kinases, with only TEC (20-fold) showing less than 30-fold selectivity for BTK. The X-ray co-crystal structure of BMS-986142 with BTK revealed that there was a narrow, deep cleft between the N- and C-lobes; the hinge residues Glu475 and Tyr476 and the gatekeeper Thr474 were located at the bottom. BMS-986142 showed good shape complementarity within the binding site. The tetrahydrocarbazole NH and the carboxamide carbonyl formed hydrogen bond interactions with the hinge residue Met477. The NH_2_ of the carboxamide formed a direct hydrogen bond with hinge Glu475 and a conserved water-mediated hydrogen interaction with Thr474. The indole carboxamide moiety was buried in the H2 pocket. The C5 toluene linker was orthogonal to the terahydrocarbazole, in the (R)-atropisomeric configuration, with the phenyl ring filling a shallow, hydrophobic subpocket (H1 pocket) formed by Gly409, Thr410 and Val416. The (S)-quinazolinedione was in turn orthogonal to the toluene linker, with the phenyl ring moiety occupying a small hydrophobic subpocket lined by the side chains of Cys481, Leu483 and Asn484. One of the quinazolinedione carbonyls engaged in a water-mediated hydrogen bond to the backbone NH of Cys481; the other carbonyl formed a water-mediated intramolecular bridging interaction with the C6 fluoro atom in tetrahydrocarbazole and was simultaneously involved in weak hydrogen bonding interactions with Gln412 and Arg525, which were mediated by water molecules. The tetrahydrocarbazole C2 dimethylcarbinol was projected toward the solvent region. The quinazolinedione and the cyclohexane fragments were both located at the opening of the deep cleft and the indole carboxamide moiety situated at the bottom, respectively, thus forming a “V” shape conformation. Contrary to the common binding mode, the DFG and A-loop residues moved far away from the binding site. In a female NZB/W lupus-prone mouse model, BMS-986142 was demonstrated to block the increase in severe proteinuria and provide protection from disease-related death. It also provided robust protection against tubulo-interstitial and glomerular nephritis, as well as inflammatory infiltration, at levels equivalent to those seen with prednisolone. In a CAIA study, administration of BMS-986142 with an oral dose of 5 mg/kg qd reduced both the severity and incidence of clinically evident paw swelling, and an oral dose of 20 mg/kg qd provided essentially complete suppression of clinical symptoms [98,99,100]. A phase 2 study in primary SS was terminated in October 2018 due to an inability to meet protocol objectives (NCT02843659). BMS-986142 completed phase 2 clinical studies in RA in May 2019 (NCT02638948), but the detailed results were not made public. The clinical trials revealed that although continuous coverage of the IC_50_ throughout the dosing interval was supported by preclinical animal studies, coverage as high as IC_90_ was required for clinical efficacy, which might pose potential safety risks [69].

## 4. Summary of Structural Information in the Ligand-Binding Site of BTK

As stated above, a large diversity of compounds has been designed to target BTK, and some of them were quite excellent inhibitors of BTK and showed high efficacy for autoimmune and inflammation diseases. All of the BTK inhibitors are postulated to bind to the active site of the BTK enzyme to exert their functions. In this section, structures of inhibitors in the co-crystal complexes with the catalytic domain of BTK were compared in order to underline key features of the protein and its binding site.

**Figure 12:**
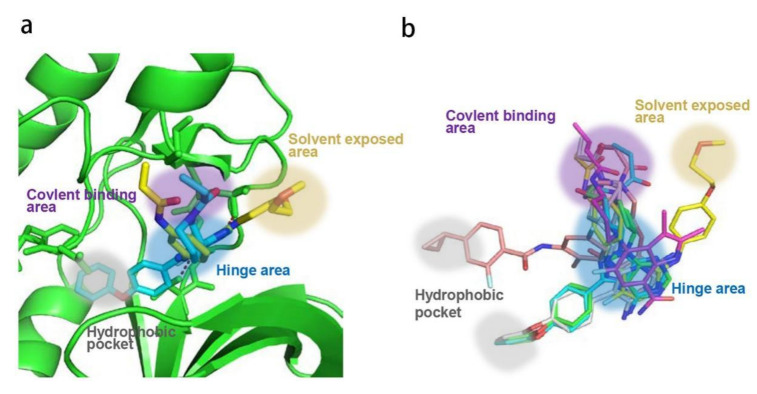
(**a**) The binding site of BTK complexed with ibrutibib (PDB code: 5P9J) is depicted with key regions highlighted by different shaded colors. The binding site is indicated in the green cartoon representation. Ibrutinib is shown as sticks and the carbon atoms are depicted in cyan. Key areas for ligand binding, such as the hydrophobic pocket (grey), covalent binding area (purple), the solvent-exposed area (light orange) and hinge area (light blue), are represented. For clarity, compound 16 from co-crystal complex (PDB code: 5P9L) was aligned to ibrutinib and its carbons are in yellow. (**b**) Superimposition of representative BTK inhibitors from crystallographic data within the binding site (Compound **1**, **8**, **16**, **15**, **17**, **18**; PDB code: 5P9J, 5P9M, 5P9L, 6O8I, 6TFP, 6OMU). Inhibitors are shown as sticks in different colors. Key areas for ligand binding are highlighted as in (**a**). All structural images were created using PyMoL [45].

It can be seen in Figure 12 that all the covalent irreversible inhibitors share a central heterocycle core and an electrophilic Michael acceptor, which develop multiple hydrogen bonds to the hinge residues and covalent interaction with the Cys481 residue, respectively. Most of these inhibitors have lipophilic moieties to bind to the hydrophobic pockets. The hydrophobic pocket of BTK has the ability to be induced to remodel in order to accommodate diverse molecular fragments. As can be seen in Figure 12b, the fluorophenylcyclopropyl moiety in remibrutinib (**17**) fit into a hydrophobic pocket that was moving away from the common position observed in most covalent inhibitors. Compared to other inhibitors, spebrutinib (**16**) lacked a lipophilic moiety, while it had a phenoxy fragment to establish additional interactions with the residues at the rim of the binding site to make up the shortfall, and the C-4′ aliphatic ether chain on the aniline simultaneously extended to the solvent.

**Figure 13:**
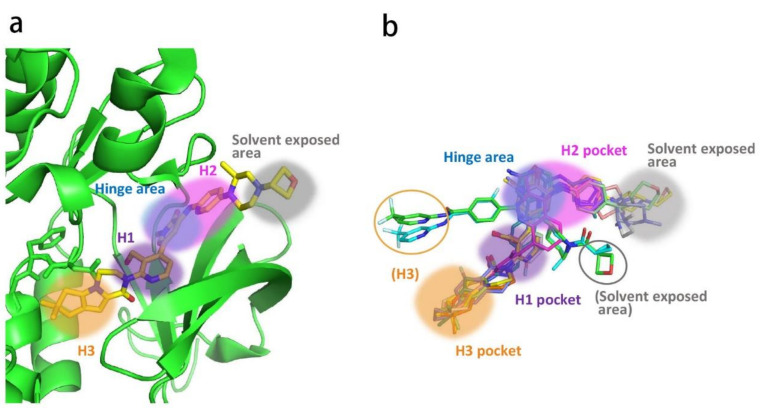
(**a**) The binding site of BTK complexed with fenebrutinib (PDB code: 5VFI) is depicted with key regions highlighted by different shaded colors. The binding site is indicated in the green cartoon representation. Fenebrutinib (34) is shown as sticks and the carbon atoms are depicted in yellow. Key areas for ligand binding, such as the H1 pocket (purple), H2 pocket (magenta), H3 pocket (orange), the solvent-exposed area (grey) and hinge area (light blue), are represented. (**b**) Superimposition of representative BTK inhibitors from crystallographic data within the binding site (Compound **2**, **24**, **25**, **28**, **30**, **31**, **32**, **33**, **34**, **35**; PDB code: 3OCS, 5FBN, 5FBO, 4Z3V, 4OTF, 4RX5, 5P9G, 5VGO, 5VFI, 6W07). Inhibitors are shown as sticks in different colors. Key areas for ligand binding are highlighted as in (**a**). All structural images were created using PyMoL [45].

Most of the non-covalent reversible inhibitors consist of four parts that, respectively, correspond to the H1 pocket, H2 pocket, H3 pocket and solvent-exposed area of the BTK binding site (Figure 13). It can be seen in Figure 13b that the trifluoromethylpyridine moieties in compounds **24** and **25** occupied the hydrophobic H3 pocket that was moving away from the common position observed in most inhibitors of this type, and the solvent-exposed moiety also shifted slightly. Compared with other inhibitors, compound **28** lacked the solvent-exposed moiety and only partly occupied the H3 pocket.

**Figure 14:**
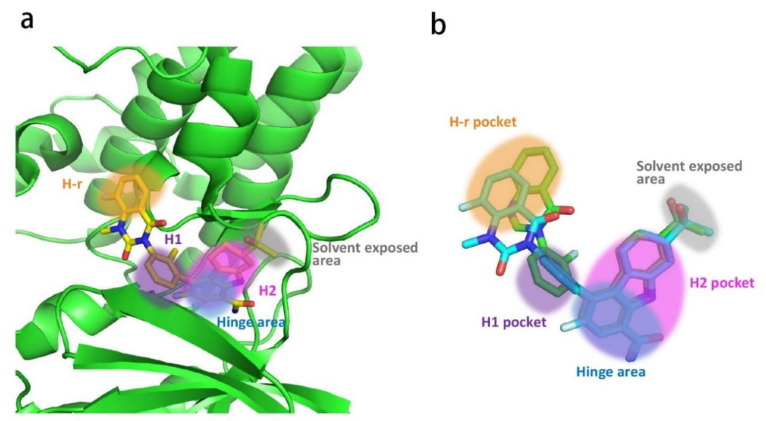
(**a**) The binding site of BTK complexed with BMS-986142 (41) (PDB code: 5T18) is depicted with key regions highlighted by different shaded colors. The binding site is indicated in the green cartoon representation. BMS-986142 is shown as sticks and the carbon atoms are depicted in yellow. Key areas for ligand binding, such as the H1 pocket (purple), H2 pocket (magenta), H-r pocket (orange), the solvent-exposed area (grey) and hinge area (light blue), are represented. (**b**) Superimposition of representative BTK inhibitors from crystallographic data within the binding site (Compound 39 and 41; PDB code: 5JRS and 5T18). Inhibitors are shown as sticks in different colors. Key areas for ligand binding are highlighted as in (**a**). All structural images were created using PyMoL [45].

In compounds **39** and **41**, the carbazole carboxamide moieties were both bound to the hinge area at the bottom of the deep, narrow cleft, with the central phenyl rings occupying a shallow hydrophobic H1 pocket (Figure 14). The phenyl rings at one end of the two molecules occupied the hydrophobic H-r pocket at the rim of the cleft and the cyclohexane or the phenyl ring at the other end were accommodated in the large H2 pocket that extended to the rim of cleft. Therefore, the two inhibitors adopted a “V” shape conformation.

The residues within 4 Å distance of ligands in the binding sites were also analyzed. It had been observed that crystallographic water was frequently involved in the protein–ligand interactions through a bridging function to increase inhibitory potency. The residues that were more than 4 Å away, but that had water-mediated hydrogen bonds with ligands, were also taken into consideration. In the six co-crystal complexes with covalent inhibitors, there were, in total, 34 residues engaged in contact with the ligands, among which eight residues were found in all six inhibitors, and three residues recurred in five inhibitors. Eleven residues were defined as high-frequency residues, namely Leu408, Val416, Ala428, Thr474, Glu475, Tyr476, Met477, Cys481, Asn484, Arg525 and Leu528. In the 12 complexes with non-covalent, reversible BTK inhibitors, a total of 43 residues were involved in the contacts with ligands; the recurring presence of 13 residues was demonstrated, namely Leu408, Thr410, Gly411, Val416, Ala428, Lys430, Thr474, Glu475, Tyr476, Met477, Gly480, Leu528 and Asp539. Eight high-frequency residues, including the glycine-rich loop residues Leu408 and Val416, the gatekeeper residue Thr474, the hinge residues Glu475, Tyr476 and Met477, as well as Ala428 and Leu528, were found in both the covalent and non-covalent inhibitor–BTK complexes. These highly conserved residues established an essential hydrophobic pocket to accommodate the heterocycle core presented in all inhibitors.

**Figure 15:**
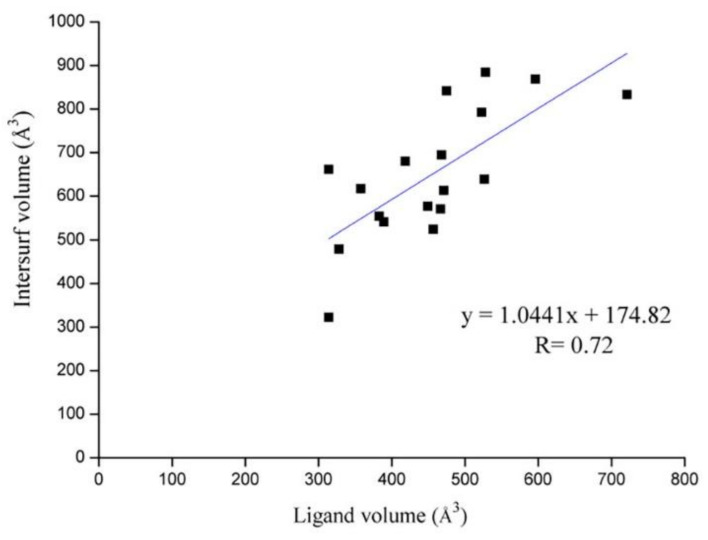
Correlation between ligand volumes and intersurf volumes in BTK–inhibitor complex structures. The ligand and intersurf volumes were calculated using UCSF Chimera [102] and the correlation coefficient is indicated.

It can usually be seen that a ligand does not fully fill the binding cavity in a complex; thus, the volume values obtained through direct calculation of the cavity cannot reflect the true binding status. Intersurf refers to an interface surface that functions as a contact surface dividing the space between proteins, or between proteins and ligands. The intersurf volume within the contact surface range would be a better parameter to account for shape complementarity [101,102]. The analysis of the eighteen BTK inhibitors herein revealed the diversity of the molecular size and shape. The volume of the binding cavities also varied greatly according to the bound ligands, ranging from 322 Å^3^ (PDB code: 4Z3V) to 884 Å^3^ (PDB code: 5P9G). A moderate but significant correlation was found between the molecular volumes of ligands and intersurf volumes within the cavities (Figure 15; correlation coefficient of 0.72), demonstrating the relationship between these two parameters. The result implies that the ligand binding domain of BTK achieves a high degree of structural plasticity by adjusting its cavity volume to accommodate ligands with different sizes.

## 5. Conclusions and Outlooks

Taken together, though BTK inhibitors differ in their chemical structures, they share some common key features. All ligands comprise an N-containing heterocycle core for binding to the hinge region and the gatekeeper residue, which simultaneously forms multiple interactions with the hydrophobic pocket around the hinge. For most of the inhibitors, the amino- or carboxamide-substituted monoheterocycle or fused bicyclic heterocycle were found to be the preferred scaffolds. In addition to the electrophilic warhead, covalent inhibitors possess a lipophilic moiety to fill another hydrophobic pocket. The non-covalent inhibitors should have a solvent-exposed fragment and two lipophilic moieties to occupy the other two hydrophobic pockets. The correlation between the intersurf volumes and the ligand volumes of cavities, as well as the good ligand–protein shape complementarity within the cavities, underlined the plasticity of the cavities, which can accommodate small molecules of different sizes and with diverse chemical structures.

A variety of BTK inhibitors has been developed for the treatment of inflammatory and autoimmune disorders, including RA, SS, MS, SLE, urticaria, pemphigus, ITP, RD, etc. Some diseases, such as RA, SS, MS, and urticaria, which are quite different from hematological malignancies, are usually non-life-threatening and long-term diseases that call for safe therapies without unexpected off-target toxicities. Therefore, researchers generally agree that reversible inhibitors are more suitable than irreversible ones, both in theory and in practice, for the treatment of these diseases [66,67,81,91,98]. In other words, the weak, non-covalent binding of reversible inhibitors with the target may reduce the toxicity and the safety risks involved with covalent, irreversible ones, thus fulfilling the treatment needs of long-term administration. A number of reversible BTK inhibitors have been designed and synthesized; some inhibitors showed single-digit nanomolar to subnanomolar IC_50_ values and excellent kinase selectivity. However, in spite of the great medicinal chemistry efforts, only a limited number of candidates have progressed to the clinical stage at present. The two reversible BTK inhibitors at the phase 2 clinical stage, fenebrutinib and BMS-986142, still seem to encounter the problem of insufficient potency.

In comparison with reversible inhibitors, the development of covalent, irreversible BTK inhibitors has progressed more rapidly. Indeed, covalent, irreversible inhibition increases the risk of off-target reactivity to biomolecules, which potentially results in immunotoxicity mutagenicity, and hepatotoxicity, as had been demonstrated in ibrutinib treatment. Nevertheless, the permanent covalent binding makes irreversible inhibitors more efficient and robust against PK liabilities than non-covalent inhibitors. Moreover, the increased efficiency would also favor the use of lower doses and a decrease in side effects. Their remarkable advantages over non-covalent inhibitors, and an improved understanding of the potential risks, inspired the development of irreversible BTK inhibitors. A knowledge of the covalent modification mechanism of inhibitors facilitated the rational design of covalent inhibitors with increased selectivity and efficacy, prolonged duration of action, as well as reduced off-target risk. The rational design of a covalent inhibitor requires a combination of two essential factors in the same molecule; namely, binding and reactivity [63]. As for the BTK inhibitor, the combination of a highly selective reversible binding scaffold and an electrophilic warhead with relatively low activity would endow the inhibitor with high potency and selectivity. Further optimization to improve the profile and stability of PK is generally required. The design strategy was well validated by the development of branebrutinib (**15**), which was a highly potent and selective, covalent, irreversible BTK inhibitor that showed rapid target inactivation in vivo, following a very low dose. Branebrutinib and some covalent BTK inhibitors, including orelabrutinib, evobrutinib, tirabrutinib, and remibrutinib, are all involved in phase 2 clinical trials for various inflammatory and immunological diseases at present. Tolebrutinib and evobrutinib are currently being studied in phase 3 clinical trials to evaluate their efficacy on MS. Specifically, tolebrutinib achieved its primary endpoint, remarkably reduced disease activity in MS patients, and was well tolerated in a phase 2b trial.

Another strategy to acquire enhanced selectivity and prolonged residence times is to design inhibitors that form reversible covalent bonds with the Cys481 residue in the binding pocket of BTK, and temporarily inactivate it. Incorporation of a cysteine-reactive cyanoacrylamide electrophile instead of acrylamide or alkynyl amide in the molecules afforded a series of reversible covalent BTK inhibitors. This class of inhibitors was highly potent and selective, and showed prolonged and tunable residence times as well as fewer unwanted off-target effects relative to those obtained with irreversible, covalent counterparts. It seems that the reversible covalent inhibitors combine the advantages of the covalent and non-covalent binding mechanisms. As a covalent, reversible inhibitor, rilzabrutinib was designated as an orphan drug for ITP and pemphigus vulgaris in the U.S. and is currently undergoing phase 3 clinical trials for both diseases.

In summary, BTK has become an important target for inflammatory and autoimmune disorders. The preclinical and clinical data demonstrated the efficacy and safety of both covalent and non-covalent small-molecule BTK inhibitors. The encouraging recent progress of both reversible and irreversible covalent inhibitors in clinical trials showed that the application of BTK-targeted therapy for inflammatory or autoimmune indications is on the horizon.

## Figures and Tables

**Table 1:** The approved BTK inhibitors and their indications.

Compound Number and Common Name	Trade Name	Indications	Dosage	Corporation	Stages
**1** (Ibrutinib)	Imbruvia	MCL, CLL, SLL, WM, cGVHD, etc	420 or 560 mg/day, capsule or tablet	Pharmacyclics/Johnson	Approved (FDA: November 2013; EMA: 2014.4; JP PMDA: March 2016)
**5** (Zanubrutinib) [4]	Brukinsa	MCL	80 mg bid, capsule	BeiGene	Approved (FDA: November 2019; CN NMPA: June 2020)
**7** (Acalabrutinib)	Calquence	MCL, CLL, SLL	100 mg bid, capsule	Acerta Pharma	Approved (FDA: November 2017)
**8** (Tirabrutinib/ONO/GS-4059)	Velexbru	PCNSL	480 mg qd, tablet	Ono Pharma	Approved (JP PMDA: 25 March 2020)
**11** (Olmutinib/HM-61713/BI-1482694)	Olita	NSCLC	800 mg/day, tablet	Hanmi Pharmaceuticals	Approved (Korea: May 2016)
**19** (Orelabrutinib/ICP-022)		MCL, CLL, SLL	150 mg qd, tablet	InnoCare Pharma	Approved (CN NMPA: December 2020)

Note: MCL, mantle cell lymphoma; CLL, chronic lymphocytic leukemia; SLL, small lymphocytic lymphoma; WM, Waldenstrom’s macroglobulinemia; cGVHD, chronic graft-versus-host disease; PCNSL, primary central nervous system lymphoma; NSCLC, non-small cell lung cancer; FDA, Food and Drug Administration; EMA, European Medicines Agency; NMPA, National Medical Products Administration; PMDA, Pharmaceuticals and Medical Devices Agency.

## Data Availability

Not applicable.

## Supplementary Materials

The following are available online, Table S1: BTK inhibitors in the development for the treatment of inflammatory and autoimmune diseases.

## Abbreviations

AIA	Adjuvant-Induced Arthritis
BCR	B-cell receptor
BTK	Bruton’s tyrosine kinase
cGVHD	Chronic graft versus host disease
CAIA	Collagen antibody-induced arthritis
CIA	Collagen-induced arthritis
CLL	Chronic lymphocytic leukemia
COVID-19	Coronavirus Disease 19
DAMA	Damage-associated molecular patterns
EAE	Experimental autoimmune encephalomyelitis
EGFR	Epidermal growth factor receptor
hPBMC	Human Peripheral Blood Mononuclear Cell
hWB	Human whole blood
IC	Immune complexes
MCL	Mantle cell lymphoma
MS	Multiple sclerosis
RRMS	Relapsing–Remitting Multiple Sclerosis
NTN	Nephrotoxic nephritis
NSCL	Non-Small Cell Lung Cancer
PAMA	Pathogen-associated molecular patterns
PCA	Passive cutaneous anaphylaxis
PD	Pharmacodynamics
PH	Pleckstrin homology domain
PK	Pharmacokinetics
RA	Rheumatoid arthritis
RMS	Relapsing multiple sclerosis
SAR	Structure–activity relationship
SH2/SH3	Src homology 2/3 domain
SLE	Systemic lupus erythematosus
SLL	Small lymphocytic lymphoma
SYK	Spleen tyrosine kinase
TCIs	Targeted covalent inhibitors
TH	Tec homology domain
TK	Tyrosine kinase domain
TLRs	Toll-like receptors
WM	Waldenström’s macroglobulinemia
