# Supplementary material for: Recent Advances in BTK Inhibitors for the Treatment of Inflammatory and Autoimmune Diseases
Source: Molecules. 2021 Aug 13;26(16):4907. doi: 10.3390/molecules26164907 (PMC8399599; doi:10.3390/molecules26164907)
Supplement: Supplementary file 1 [file molecules-26-04907-s001.zip › molecules-1330752-supplementary.pdf]

Support Information

Recent Advances in BTK Inhibitors for the Treatment of Inflammatory and Autoimmune Diseases

Datong Zhang [1,*], He Gong [1] and Fancui Meng [2]

[1] School of Chemistry and Chemical Engineering, Qilu University of Technology (Shandong Academy of Sciences), 3501 Daxue Road, Jinan 250353, China; gonghedeyouxiang@163.com

[2] Tianjin Key Laboratory of Molecular Design and Drug Discovery, Tianjin Institute of Pharmaceutical Research, 306 Huiren Road, Tianjin 300301, China; mengfc@tjipr.com

* Correspondence: dtzhang@qlu.edu.cn

Table S1. BTK inhibitors in the development for the treatment of inflammatory and autoimmune diseases.

Compound Number and Common Name	Type	BTK IC_{50} （nM）	Animal Model	Progress in the Treatment of Inflammatory and Autoimmune Diseases
**1** (Ibrutinib)	Irreversible	0.5	CIA, CAIA	Phase 2, SARS-CoV-2 and Pulmonary Injury; capsule
**2** (CGI-1746)	Reversible	1.9	CIA, CAIA	Preclinical
**3** (Rilzabrutinib)	Reversible, covalent	1.3	CIA	Phase 3, ITP; 400 mg bid po<br>Phase 2, RD; 400 mg bid po, plus glucocorticoids<br>Phase 3, pemphigus
**4**	Irreversible	1	CIA	Preclinical
**5** (Zanubrutinib)	Irreversible	1.8	NA	Phase 2, RD; 80 mg bid po,<br>Phase 2, lupus nephritis; 40 or 160 mg bid po or 160 mg qd po
**6**	Irreversible	21.7	CIA	Preclinical
**7** (Acalabrutinib)	Irreversible	3	NA	Phase 2, RA; 15 mg qd po, plus methotrexate
**8** (Tirabrutinib)	Irreversible	6.8	CIA	Phase 1, RA; 10 mg bid or 20 mg qd po<br>Phase 2, SS; 40 mg qd po<br>Phase 2, pemphigus
**9** (Tolebrutinib)	Irreversible	0.7	EAE	Phase 3, MS; oral administration

**10**	Irreversible	2.1	CIA	Preclinical
**11 (**Olmutinib)	Irreversible	13.9	NA	Preclinical
**12**	Irreversible	29.9	NA	Preclinical
**13** (Poseltinib)	Irreversible	1.95	CIA, NZB/W, MRL/Ipr	Phase 2, RA; 5 mg, 10mg or 30 mg qd po
**14**	Irreversible	0.46	NA	Preclinical
**15** (Branebrutinib)	Irreversible	0.1	CIA, NZB/W F1	Phase 2, SLE or SS or RA; oral administration
**16** (Spebrutinib)	Irreversible	< 0.5	CIA	Phase 2, RA; 375 mg/day, po
**17** (Remibrutinib)	Irreversible	1.3	CIA	Phase 2, SS; 10 mg/day, 25 or 50 mg/day, po<br>Phase 2, urticaria; oral administration
**18** (Evobrutinib)	Irreversible	8.9	CIA, NZB/W,	Phase 3, MS; oral administration<br>Phase 2, RA; 25 mg or 75 mg qd po or 50 mg bid po
**19** (Orelabrutinib)	Irreversible	1.6	NA	Phase 1/2, SLE; 50 mg, 80 mg or 100 mg, qd po<br>Phase 2, MS; oral administration
**20** (BI-BTK-1)	Irreversible	0.9	NTN, NZB/W, MRL/Ipr	Preclinical
**21**	Irreversible	1.93	CIA	Preclinical
**22**	Reversible, covalent	4	NA	Preclinical
**23**	Reversible, covalent	1.9	NA	Preclinical
**24**	Reversible	0.27	NA	Preclinical
**25**	Reversible	0.31	CIA	Preclinical
**26**	Reversible	0.1	CIA	Preclinical
**27**	Reversible	3	CIA	Preclinical
**28**	Reversible	4	CIA	Preclinical
**29**	Reversible	5.3	CIA	Preclinical
**30** (GDC-0834)	Reversible	6	CIA	Preclinical
**31**	Reversible	3	NA	Preclinical
**32** (RN-486)	Reversible	4	CIA, CAIA, PCA, AIA	Preclinical
**33** (G-744)	Reversible	2	CIA, NZB/W F1	Preclinical
**34** (Fenebrutinib)	Reversible	0.91(Ki)	CIA	Phase 2, RA; 200 mg bid po<br>Phase 2, SLE; 150 or 200 mg bid po<br>Phase 2, urticaria; 200 mg bid po

**35** (BIIB068)	Reversible	1	TI-2 antigen mouse	Phase 1, SLE; oral administration
**36**	Reversible	0.3	PCA	Preclinical
**37**	Reversible	0.39	CIA, PCA	Preclinical
**38** (BMS-935177)	Reversible	3	CIA, CAIA	Preclinical
**39**	Reversible	4	NA	Preclinical
**40** (BMS-986143)	Reversible	0.26	CIA, CAIA	Preclinical
**41** (BMS-986142)	Reversible	0.5	CAIA, NZB/W F1	Phase 2, RA; oral administration

Note: (a) Some clinical trial do not indicate dosage forms and dose of the drug. (b) SARS-CoV-2, severe acute respiratory syndrome coronavirus-2; MS, multiple sclerosis; SLE, systemic lupus erythematosus; ITP, immune thrombocytopenia; RD, IgG4-related disease; RA, rheumatoid arthritis; SS, Sjogren's syndrome; CIA, collagen-induced arthritis; CAIA, collagen antibody-induced arthritis; EAE, experimental autoimmune encephalomyelitis; NTN, nephrotoxic nephritis. PCA, passive cutaneous anaphylaxis; AIA, Adjuvant-induced Arthrits. NA, not available.
